# RAB3A-mediated BAG6 translocation promotes non-small cell lung cancer tumorigenesis and progression

**DOI:** 10.1007/s13402-025-01123-z

**Published:** 2025-10-22

**Authors:** Xiaoli Liu, Wen Wang, Wanwei Cao, Zhanyu Li, Hao Huang, Fang Liu, Yang Wang, Zhijuan Zhong, Hongyu Zhang, Xiaofeng Pei, Hongtao Chen

**Affiliations:** 1 Department of Oncology, The Fifth Affiliated Hospital of Sun Yat-sen University, Zhuhai, 519000 China; 2 Guangdong Provincial Engineering Research Center of Molecular Imaging, The Fifth Affiliated Hospital of Sun Yat-sen University, Zhuhai, 519000 China; 3 Department of Pathology, The Fifth Affiliated Hospital of Sun Yat-sen University, Zhuhai, 519000 China; 4 Department of Laboratory, The Fifth Affiliated Hospital of Sun Yat-sen University, Zhuhai, 519000 China; 5 Department of Laboratory, Xinjiang Medical University Affiliated Second Hospital, Wulumuqi, 830028 China

**Keywords:** Non-small cell lung cancer, RAB3A, BAG6, Mitophagy, p53/Rb signaling, Cisplatin

## Abstract

**Purpose:**

RAB3A, a member of the RAS oncogene family, plays a central role in regulated exocytosis and secretion. However, its function, molecular mechanism, and clinical significance in non-small cell lung cancer(NSCLC) remain largely undetermined.

**Methods:**

We analyzed the expression of RAB3A and its correlation with overall survival using multiple databases and immunohistochemistry staining. The oncogenic role of RAB3A in NSCLC was investigated through various cellular assays, including cell viability and colony formation assays. Protein mass spectrometry experiment, immunoprecipitation, immunofluorescence, subcellular fractionation, mitochondria isolation, cycloheximide assays, and lung cancer xenograft mouse models were performed to clarify the molecular mechanism of RAB3A in NSCLC progression.

**Results:**

The expression of RAB3A was upregulated in NSCLC patients and high level of RAB3A correlated with a poor overall survival. RAB3A depletion inhibited the cell proliferation. Mechanistically, RAB3A knockdown enhanced the nuclear translocation of BAG6-EP300, leading to the acetylation of p53 and Rb. This acetylation strengthened the p53/Rb signaling pathway, thereby suppressing NSCLC progression. Additionally, RAB3A facilitated the transportation of BAG6 to the mitochondria, promoting mitophagy and increasing resistance to cisplatin in NSCLC cells.

**Conclusion:**

Our findings elucidate the role and underlying mechanisms of RAB3A in NSCLC progression. Thus, RAB3A emerges as a potential prognosis prediction biomarker and therapeutic target for NSCLC patients.

**Clinical trial number:**

Not applicable.

**Supplementary Information:**

The online version contains supplementary material available at 10.1007/s13402-025-01123-z.

## Introduction

Lung cancer is the leading cause of cancer-related deaths worldwide, with high morbidity and mortality in China [1]. Non-small cell lung cancer (NSCLC) accounts for 85% of lung cancer cases [2]. Significant progress has been made in NSCLC treatment, including chemotherapy, radiotherapy, and immunotherapy [3, 4]. However, due to the lack of effective early-stage diagnostic methods, the majority of NSCLC patients are diagnosed at advanced stages, resulting in a 5-year survival rate of approximately 20% [5, 6]. Therefore, there is a pressing necessity to investigate the novel biomarkers and carcinogenic factors in NSCLC development and progression.

Increasing lung cancer driver genes have been discovered in recent years [7, 8]. As the small Ras-like GTPase, RAB family have been observed as the driving gene of cancer mediating the occurrence, metastasis, and drug resistance [9, 10]. During the last decades, several RAB protein members have been identified as oncogenes promoting the progression of lung cancer [11, 12]. Previous studies have demonstrated that RAB37 plays a role in mediating the IL-6 secretion by macrophages. Subsequently, through the RAB37/IL-6/STAT3 transcription axis, it can upregulate the expression of PD-1 in T cells, thus promoting the immune escape of lung cancer [13]. Meanwhile, TbC1D23 participates in the interaction between RAB11A and β1 integrin and activates the corresponding signal pathway promoting the occurrence of NSCLC [14]. Regarding lung cancer drug resistance, osimertinib promotes the release of exosomes by upregulating RAB17 [15]. Therefore, RAB families play a momentous influence on the progress of lung cancer.

Among the RAB family, RAB3A plays a crucial role in mediating membrane transport [16]. RAB3A binds to or dissociates from the vesicle membrane in a GTP-or GDP-bound state and plays a role in the fusion of synaptic vesicles. Recent evidence suggests RAB3A, along with its interacting proteins, contributes significantly to Ca^2+^-dependent exocytosis, with a particular emphasis on neurotransmitter release at synaptic terminals [17]. Meanwhile, it has been reported that RAB3A is involved in lysosomal trafficking to the plasma membrane [18]. Previous studies have reported that abnormally upregulated RAB3A was discovered in different tumors such as breast cancer, insulinoma, and tumors originating from nervous system [19–21]. The upregulated RAB3A has been demonstrated that is associated with high-grade gliomas, suggesting an oncogenic role for RAB3A in brain tumors [22]. Compared with other RAB family members, the research of RAB3A is lacking. Therefore, elucidating the function and molecular mechanism of RAB3A in the emergence and development of NSCLC may provide novel strategies and theoretical basis.

In this research, we explored the upregulation of RAB3A expression in NSCLC tissues and its correlation with poor prognosis. Mechanistic investigation indicated that RAB3A knockdown induced BAG6-EP300 transport to the nucleus, increasing the acetylation of p53 and Rb and inhibiting NSCLC cell proliferation and clonal growth. Interestingly, RAB3A promoted the mitochondrial transport of BAG6, inducing mitophagy under oxidative stress to enhance NSCLC tolerance to cisplatin. Therefore, our findings reveal the mechanism and clinical significance of RAB3A in NSCLC progression.

## Materials and methods

### Data sources

The Cancer Genome Atlas (TCGA) database (https://portal.gdc.cancer.gov/) served as the source for NSCLC expression profiles and pertinent clinical data.RAB3A expression in NSCLC, based on the RNA sequencing data of TCGA, GTEx and GEO (GSE19188 [23], GSE33532 [24]) project, was analyzed using the online tool Sangerbox (http://www.sangerbox.com/tool) [25] and Assistant for Cinical Bioinformatics (https://www.aclbi.com/) [26]. The Kaplan–Meier Plotter (https://kmplot.com/analysis/) was utilized to investigate the correlations between RAB3A expression and overall survival of NSCLC patients [27].

### Patients and samples

Lung tissues, including normal specimens and NSCLC tissues, were collected from NSCLC patients at the Fifth Affiliated Hospital of Sun Yat-sen University. Every patient gave their written informed consent, and this study got the approval from the Medical Research Ethics Committee of the Fifth Affiliated Hospital of Sun Yat-sen University(2023-K172-1).

### Plasmid construction

The extraction of total RNA was accomplished using Total RNA Kit I (Omega, R6834-02). Following this, reverse transcription of 1 µg RNA was performed in a 20 µL reaction volume, utilizing Hifair^®^ II 1st Strand cDNA Synthesis SuperMix (Yeasen, 11120ES60). These cDNAs tagged with Flag or HA were cloned by PCR and inserted into the pSin-EF2-puro vector. Primer sequences were as follows:

RAB3A-F: 5’-ATGGCATCCGCCACAGACTC- 3’.

RAB3A-R: 3’-GCAGGCGCAGTCCTGGT- 5’.

BAG6-F: 5’-ATGGAGCCTAATGATAGTACCAGTACCGCT- 3’.

BAG6-R: 3’-AGGATCATCAGCAAAGGCCCG- 5’.

EP300-F: 5’-ATGGCCGAGAATGTGGTGGAAC- 3’.

EP300-R: 3’-GTGTATGTCTAGTGTACTCTGTGAGAGGTTTG- 5’.

### Cell culture

The cell lines NCI-H1993, NCI-H1975 and HEK-293T were acquired from American Type Culture Collection (ATCC, Manassas, VA). Experiments exclusively utilized mycoplasma-free cells. These cells underwent cultivation in either RPMI-1640 or DMEM medium (Thermo Scientific, Waltham, MA, USA). The growth medium was augmented with 10% fetal bovine serum (Thermo Scientific, Waltham, MA, USA), along with 100 µg/ml streptomycin and 100 units/ml penicillin. Incubation conditions were maintained at 37 °C in an atmosphere containing 5% CO₂.

### Construction of cell lines with stable RAB3A expression

To stably knock down or overexpress endogenous RAB3A expression, we performed the lentiviral production as follows. The transfection protocol for HEK-293T cells involved a combination of plasmids: 3 µg of plenti-crispr-v2-sgRNA/pSin-EF2-cDNA, 2 µg of psPAX2, and 1 µg of pMD2G. This mixture was introduced into the cells using 24 µL of polyethylenimine (PEI) at a concentration of 2 mg/mL. Viral supernatants were collected after being transfected for 48 h. In six-well plates, cells were subjected to viral infection in the presence of 10 µg/mL of polybrene (Sigma). For the selection of stable cell lines, 0.5 µg/mL puromycin was employed. The sequences of small guide RNA (sgRNA) are listed as follows:

sgRAB3A#1: 5’-CGGGTGGTGTCATCAGAACG- 3’.

sgRAB3A#2: 5’-GGATGAAGCCCATAGCGCCC- 3’.

### Antibodies

Specific antibodies against RAB3A (PA1-770), E2F1 (32-1400), anti-rabbit horseradish-peroxidase (HRP)-conjugated antibody (31460), anti-mouse HRP-conjugated antibody (31430), Goat anti-rabbit Alexa Fluor-488 (A-11034), and Goat anti-rabbit Alexa Fluor-594 (A-11037) were acquired from ThermoFisher. Anti-BAG6(26417-1-AP), LC3(14600-1-AP), p62(18420-1-AP), Histone H3 (17168-1-AP), and rabbit IgG (30000-0-AP) antibodies were purchased from Proteintech. Anti-Flag (8146), anti-GAPDH(5174), anti-HA(3724), anti-Ac-K(9814), anti-V5 (13202),and anti-β-actin (3700) were purchased from Cell Signaling Technology. Anti-EP300 (ab275378), anti-p53 (ab32049), anti-Rb (ab181616), anti-TOMM20 (ab186735), anti-p21 (ab109520), anti-PINK1(ab216144) and anti-Parkin (ab77924) antibodies were sourced from Abcam.

### Immunohistochemistry staining (IHC)

The anti-RAB3A antibody (1:500; Thermo Scientific, USA) was applied and incubated at 4 °C overnight within a humidified container. Counterstaining was performed using hematoxylin. Sections were then treated with the anti-rabbit IgG secondary antibody (1:1000; Thermo Scientific, USA) and washing again. Subsequently, sections were incubated with ABC reagent followed by DAB staining. Immunohistochemical scoring was determined as previous described [28]. Slides were digitally scanned using a Pannoramic 250 FLASH III Digital Slide Scanner (P250 FLASH; A3DHISTECH Ltd.).

### Western blot (WB)

The cells got washed on three occasions with PBS and treated with RIPA lysis buffer (Beyotime, China) containing 10mM PMSF (Beyotime, China) and protease inhibitor Cocktail (Med Chem Express, USA). The Enhanced BCA Protein Assay Kit (Beyotime, China) was employed to quantify the concentration of cellular lysates. Protein separation was achieved through 10–15% SDS-PAGE, conducted at 80 V and 120 V. The proteins were then transferred onto PVDF membranes (Millipore, USA) at 350 mA. The membrane was incubated with the indicated primary antibodies followed by the incubation with HRP-conjugated secondary antibodies. Images were detected by MiniChemi910 Chemiluminescence imager (SINSAGE, China).

### Colony formation assay

NCI-H1993 and NCI-H1975 cells were seeded into 6-well plates in RPMI-1640 medium with 10% FBS to measure colony formation. The cells were incubated at 37 °C and in an environment with 5% CO_2_ for 15 days. Subsequently, they were fixed by using 4% paraformaldehyde. Thereafter, a staining procedure was performed on the cells with 0.05% crystal violet for 10 min. Photographs of the colonies were captured, and each experiment was repeated in triplicate.

### Cell viability assay

To assess cell viability, we conducted Cell Counting Kit-8 (CCK-8) assay. NCI-H1993 and NCI-H1975 cells were plated at a concentration of 5000 cells per well in 96-well plates. The cells underwent incubation with 10 µL CCK8 (Biosharp, BS350B) for 4 h, maintained at 37 °C. Subsequently, optical density measurements were obtained at 450 nm employing a microplate reader (Thermo Scientific, USA), with readings taken once every 24 h over a 3-day period.

### Immunofluorescence (IF)

Cells were seeded into glass-bottomed culture dishes (NEST, 801002). They were then washed 3times using PBS, followed by fixation for 15 min using 4% paraformaldehyde in PBS. After permeabilization with 0.5% Triton X-100 in PBS for 30 min and subsequent washing three times with PBS, the cells were incubated with 3% BSA in PBS for 1 h at room temperature. Following this, the cells were subjected to overnight incubation with primary antibodies at 4 °C. The next day, Alexa Fluor-conjugated secondary antibodies were applied to the cells for a 1-hour incubation period at room temperature. To conclude the procedure, the nuclei were stained using DAPI (Beyotime, China) diluted in PBS. The cells were imaged using laser scanning confocal microscopes (Zeiss LSM880 with Airyscan).

### Co-immunoprecipitation (Co-IP) and mass spectrometry (MS)

Cells co-transfected with tagged constructs were lysed in IP Lysis Buffer. Subsequently, the lysates were then incubated with the pre-cleared with the anti-tagged beads for 3 h at 4 °C. In the endogenous immunoprecipitation assay, pre-cleared with Protein A/G Agarose, and incubated overnight with target antibody. Complexes were captured with Protein A/G Agarose, washed, and eluted. Proteins were analyzed by western blot. Input and IgG controls were included. Immunoprecipitates were sent for mass spectrometry analysis(Guangzhou Saicheng Biotechnology).

### Cycloheximide (CHX) chase assay

NCI-H1993 and NCI-H1975 cells were transfected to knock down RAB3A. The cells were incubated with 20 ng/ml CHX (Sigma‒Aldrich, C1988) for various periods of time to prevent de novo protein synthesis. Subsequently, the cells were washed by PBS and lysed for western blot analysis.

### Subcellular fractionation

Cytosolic and nuclear extract fractionation was conducted using NE-PER™ Nuclear and Cytoplasmic Extraction Reagents (Thermo Scientific, 78833) according to the manufacturer’s instructions.

### Mitochondria isolation

Mitochondria were isolated from the NCI-H1993 and NCI-H1975 cells using Mitochondria Isolation Kit (Thermo Scientific, 89874) according to the manufacturer’s instructions.

### Animal work

The study was approved by the Animal Ethics Committee of First Affiliated Hospital of Xinjiang Medical University (IACUC-20200331-102). Female BALB/c nude mice (5-week-old, *n* = 6 per group) were obtained from BesTest Bio-Tech Co,.Ltd (Zhuhai, China) and maintained under pathogenfree conditions. NCI-H1975 cells were stably transfected with sgNC and sgRAB3A#1, respectively. Subsequently, 5 × 10^6^ cells were injected into BALB/c nude mice. The nude mice were administered intraperitoneally with cisplatin (15 mg/kg; Selleck, S1166) or normal saline three times a week starting at 14 days. Tumor weight was measured after 30 days.

### Statistical analysis

Statistical analyses were performed using SPSS 16.0 software and GraphPad Prism 8.0 software. The data are expressed as mean ± SD. Kaplan–Meier survival analysis was conducted using the log-rank test. The statistical significance of differences was evaluated through unpaired Student’s t-tests or Analysis of Variance (ANOVA) with corresponding numbers. Statistical significance was defined as *p* < 0.05.

## Results

### RAB3A is upregulated in NSCLC and correlates with a poor prognosis

**Fig. 1:**
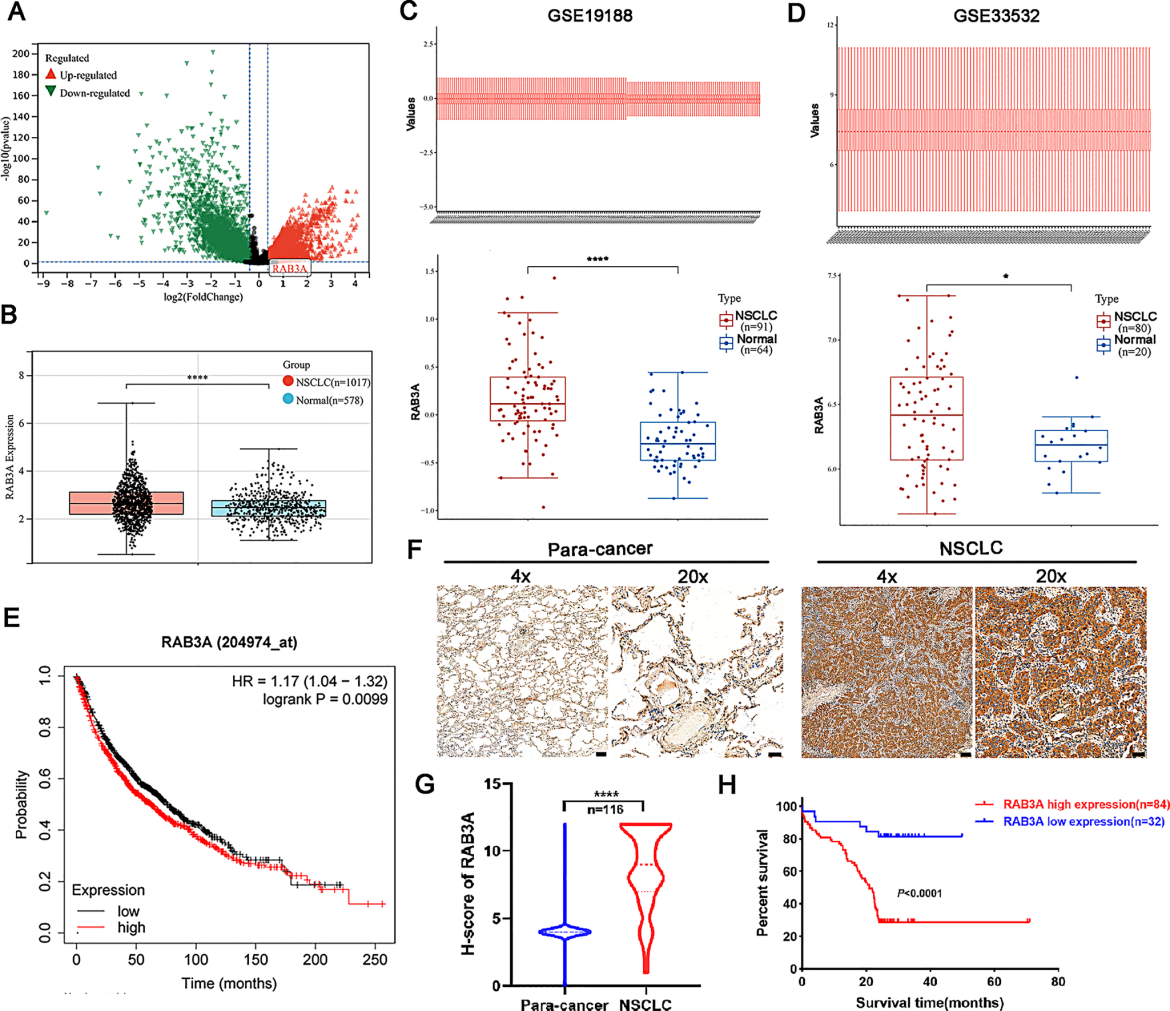
High expression of RAB3A significantly correlated with a poor prognosis for NSCLC patients. **A** Volcano plot illustrating genes significantly regulated in NSCLC tissues. **B** Expression of RAB3A in NSCLC tissues and lung tissues according to TCGA dataset. **C**, **D** Expression of RAB3A in NSCLC tissues and lung tissues according to GSE19188 and GSE33532. **E** Kaplan–Meier survival analysis of the correlation between RAB3A expression and overall survival in NSCLC cohort. **F** Representative immunohistochemical images of RAB3A in NSCLC tissues or matched adjacent tissues. Scale bars: 200 μm (4×), 50 μm (20×). **G** Statistical data of the IHC staining scores. **H** Kaplan–Meier analysis for overall survival of NSCLC patients from IHC cohort. Data are presented as the mean ± SD. The significance level was represented by * *p* < 0.05, *** *p* < 0.001, **** *p* < 0.0001

Analysis of RNA-seq data from TCGA NSCLC indicated significant differences in gene expression between NSCLC tissues and normal lung tissues (Fig. 1A). Notably, the expression of RAB3A was found to be substantially higher in NSCLC tissues compared to normal lung tissues (Fig. 1B). To further investigate the expression of RAB3A in NSCLC, we analyzed the GEO database, including GSE19188 and GSE33532. The results confirmed that RAB3A expression levels were markedly elevated in NSCLC tissues relative to normal lung tissues (Fig. 1C and D). Additionally, the Kaplan-Meier Plotter database in NSCLC revealed that high expression of RAB3A was associated with worse overall survival (Fig. 1E). To verify the results of the database, we performed immunohistochemistry assay to assess RAB3A expression in 116 NSCLC cases and their adjacent tissues. As illustrated in Fig. 1F and G, RAB3A protein levels were significantly higher in NSCLC tissues. According to the cut-off value of RAB3A H-score, the NSCLC patients were classified into high and low RAB3A expression groups. The analysis revealed that patients with high RAB3A expression exhibited poorer overall survival compared to those with low RAB3A expression (Fig. 1H). Additionally, high RAB3A expression was significantly correlated with key clinical parameters, including tumor size (*p* = 0.024) and lymph node metastasis (*p* = 0.002) (Table 1). Overall, these findings suggest that RAB3A upregulation in NSCLC tissues is correlated with a poor prognostic outcome.

**Table 1:** Relationship between expression of RAB3 and clinicopathologic features of NSCLC patients (*n* = 116)

Clinical pathologic characteristics	*n*	RAB3A		*p* value
		High (%)	Low (%)	
Gender				
Female	63	43(51.19)	20(62.50)	0.274
Male	53	41(48.81)	12(37.50)	
Age(years)				
≥ 60	57	42(50.00)	15(46.88)	0.763
< 60	59	42(50.00)	17(53.13)	
Tumor Size(cm)				
≥ 5	23	21(25.00)	2(6.25)	0.024*
< 5	93	63(75.00)	30(93.75)	
Lymph node metastasis				
No	95	63(75.00)	32(100.00)	0.002**
Yes	21	21(25.00)	0(0.00)	
TNM Stage				
Stage Ⅰ-Ⅱ	96	66(78.57)	30(93.75)	0.053
Stage Ⅲ-Ⅳ	20	18(21.43)	2(6.25)	

Abbreviation: TNM, Tumor Node Metastasis.* *p* < 0.05 ** *p* < 0.01

### RAB3A knockdown in NSCLC cells inhibits their proliferation

**Fig. 2:**
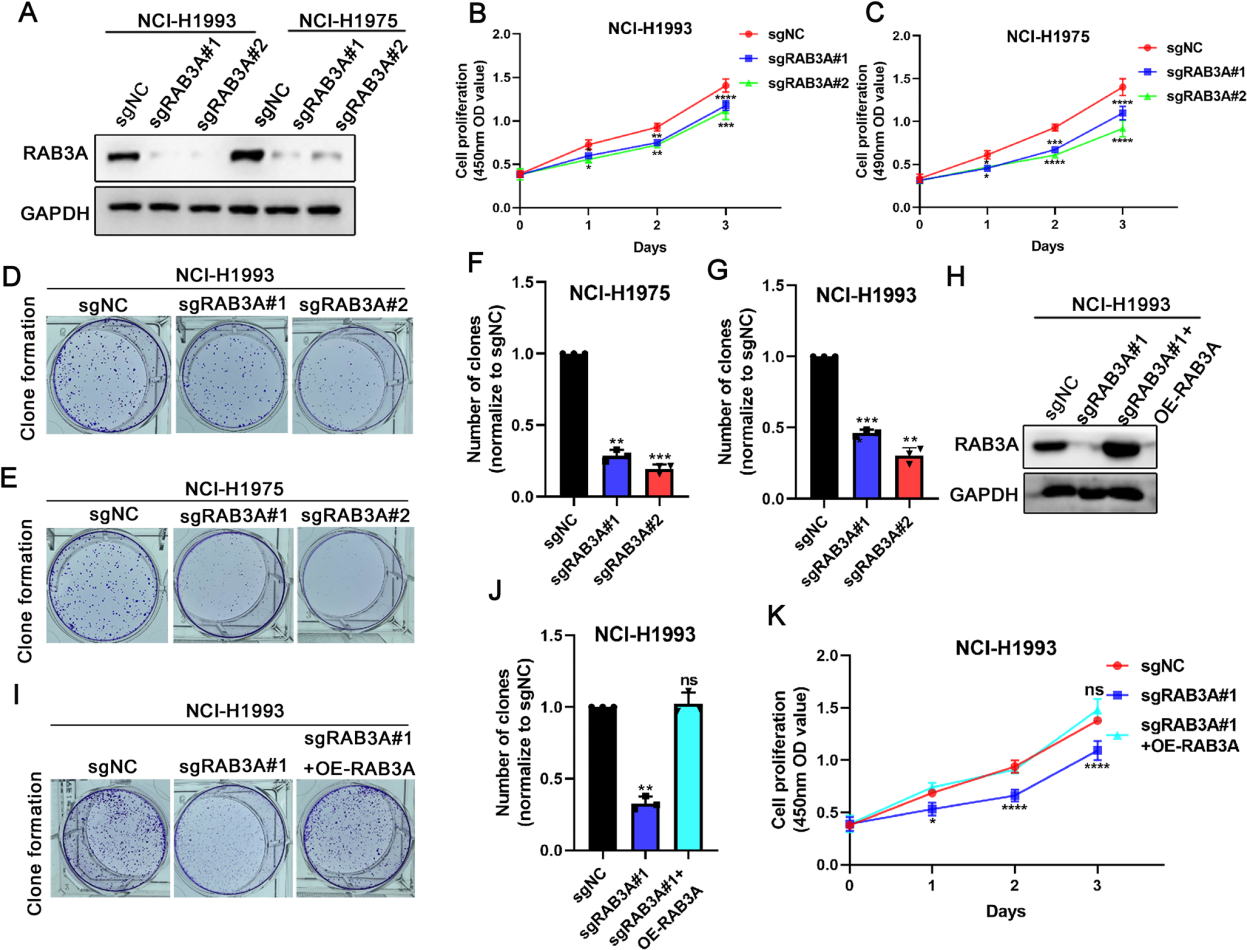
RAB3A promotes the proliferation of NSCLC cells. **A** Western blot analysis of RAB3A proteins in the indicated stable cells. **B**,** C** CCK8 assay of cell viability in the indicated stable NCI-H1993 and NCI-H1975 cells in three independent experiments. **D-G** Colony formation assay in the indicated stable NCI-H1993 and NCI-H1975 cells. **H** Western blot analysis of RAB3A proteins in reconstituted cells. **I**,** J** Colony formation assay in reconstituted cells. **K** CCK8 assay of cell viability in reconstituted cells. Error bars represent the mean ± SD. The significance level was represented by *p < 0.05, **p < 0.01, ***p < 0.001, **** *p* < 0.0001. ns, nonsignificant difference

To determine the potential biological function of RAB3A in NSCLC, we employed two pairs of small guide RNAs targeting RAB3A sequences to knock down RAB3A in NCI-H1993 and NCI-H1975 cells (Fig. 2A). Next, we conducted CCK8 assay and found that the RAB3A knockdown inhibited the proliferation of both NCI-H1993 and NCI-H1975 cells (Fig. 2B and C). Consistently, the data of colony formation assay revealed that RAB3A knockdown restrained their clonogenicity (Fig. 2D-G). To exclude potential confounding from incomplete editing, we established RAB3A-null monoclonal clones (Fig. S1A). RAB3A knockout led to > 40% suppression of proliferation and > 80% reduction in clonogenicity (Fig. S1B and C). To mitigate potential off-target effects of the sgRNAs, we performed a rescue experiment by reintroducing RAB3A after its knockdown in NCI-H1993 cells (Fig. 2H). Subsequent colony formation and CCK8 assays revealed that the clonogenicity and proliferation ability were restored in NCI-H1993 cells upon silencing RAB3A and concurrent overexpression of RAB3A (Fig. 2I-K). To model physiologically relevant rescue, RAB3A was reconstituted in deficient NCI-H1993 cells across expression gradients (Fig. S2A). Notably, restoration to near-basal levels significantly rescued proliferation and colony formation deficits compared with the RAB3A-null group (Fig. S2B and C). Higher expression (high-titer) provided no significant enhancement over the medium-titer rescue, that physiological RAB3A levels are sufficient for oncogenic function. Therefore, these results demonstrate that RAB3A plays crucial roles in regulating the proliferation of NSCLC cells.

### RAB3A interacts and colocalizes with BAG6

**Fig. 3:**
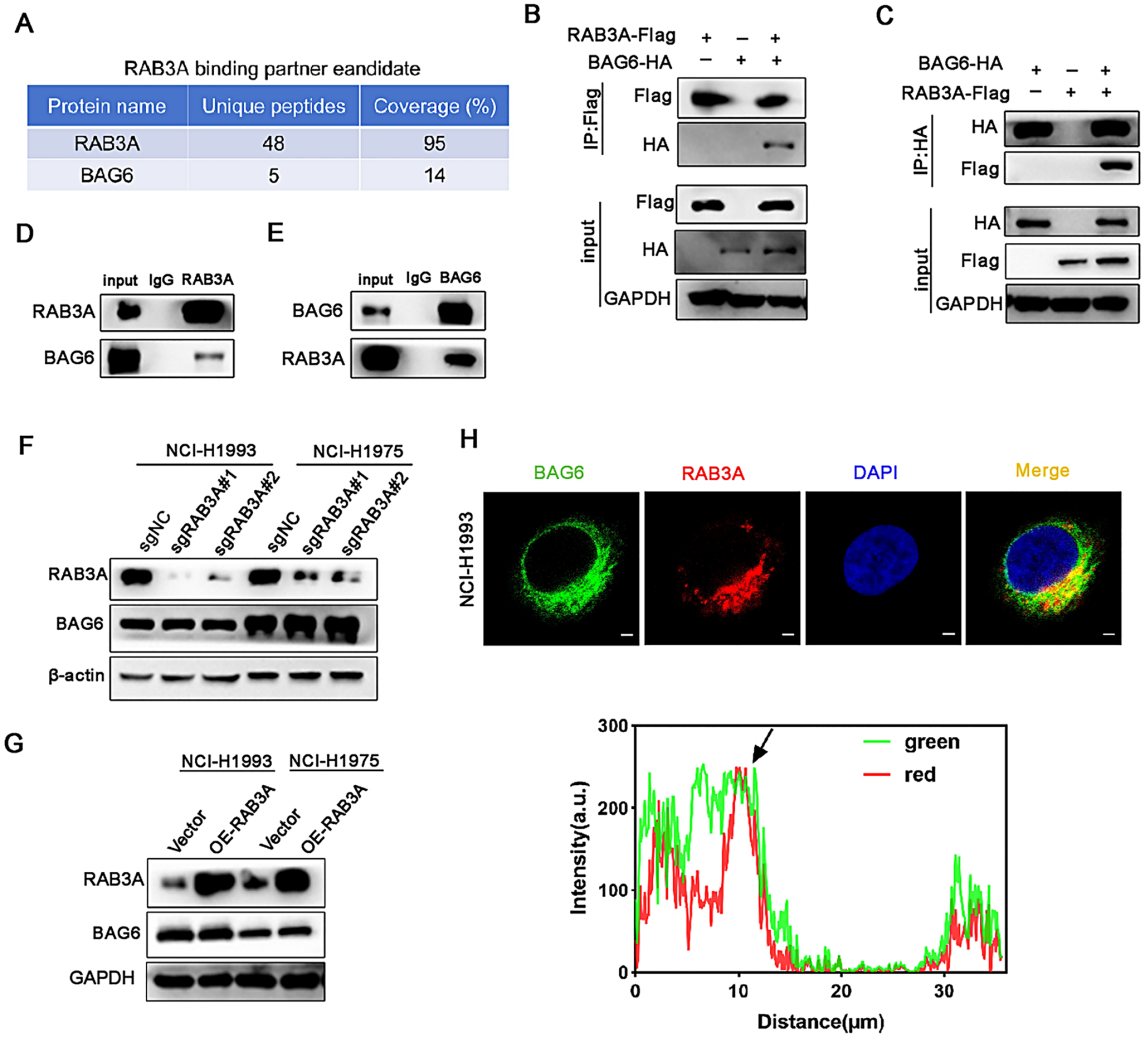
Co-localization exists for RAB3A and BAG6. **A** Protein mass spectrometry analysis of RAB3A binding partner candidates in the indicated stable cells. **B**,** C** Western blot analysis of the interaction of RAB3A and BAG6 in HEK-293T cells co-transfected with the indicated plasmids. **D**,** E** Immunoprecipitation assay showing the interaction between endogenous RAB3A and BAG6. IgG was used as a control. **F** Western blot analysis of BAG6 expression using NCI-H1993 and NCI-H1975 cells with sgRNAs targeting RAB3A. **G** Western blot analysis of the BAG6 levels in the indicated stable cells. **H** Immunofluorescence analysis of the co-localization between RAB3A and BAG6. Scale bars: 5 μm

To further establish how RAB3A promotes tumor growth, we conducted a protein mass spectrometry experiment (MS) for examining the interaction between RAB3A and specific molecules. We detected BAG6, which is a nuclear protein of 150 kDa participates that binds to RAB3A (Fig. 3A). Bcl-2-associated athanogen-6(BAG6) plays an important role in EP300 intracellular localization, mitophagy and antigen presentation [29, 30]. To assess the interaction between RAB3A and BAG6, we co-transfected RAB3A-Flag and BAG6-HA plasmids into HEK-293T cells. Reciprocal coimmunoprecipitation experiments revealed that there was an interaction between exogenous BAG6 and RAB3A (Fig. 3B and C). Subsequently, we performed endogenous immunoprecipitation assay and further proved the endogenous interaction between RAB3A and BAG6 (Fig. 3D and E). We knocked down or overexpressed RAB3A in NCI-H1993 and NCI-H1975 cells and detected the protein levels of BAG6. Interestingly, the results suggested that the protein levels of BAG6 remained unchanged after RAB3A knockdown or overexpression (Fig. 3F and G). Furthermore, immunofluorescence assay showed that BAG6 colocalized with RAB3A (Fig. 3H). To investigate clinical consistency with cell line observations, we analyzed the subcellular localization of BAG6 in NSCLC specimens stratified by RAB3A expression. Double-immunohistochemistry analysis demonstrated cytoplasmic co-localization of BAG6 and RAB3A in RAB3A-high tumors(Fig. S3). Collectively, these data indicate that RAB3A regulates the subcellular localization of BAG6.

### RAB3A knockdown promotes BAG6-EP300 translocation into the nucleus

**Fig. 4:**
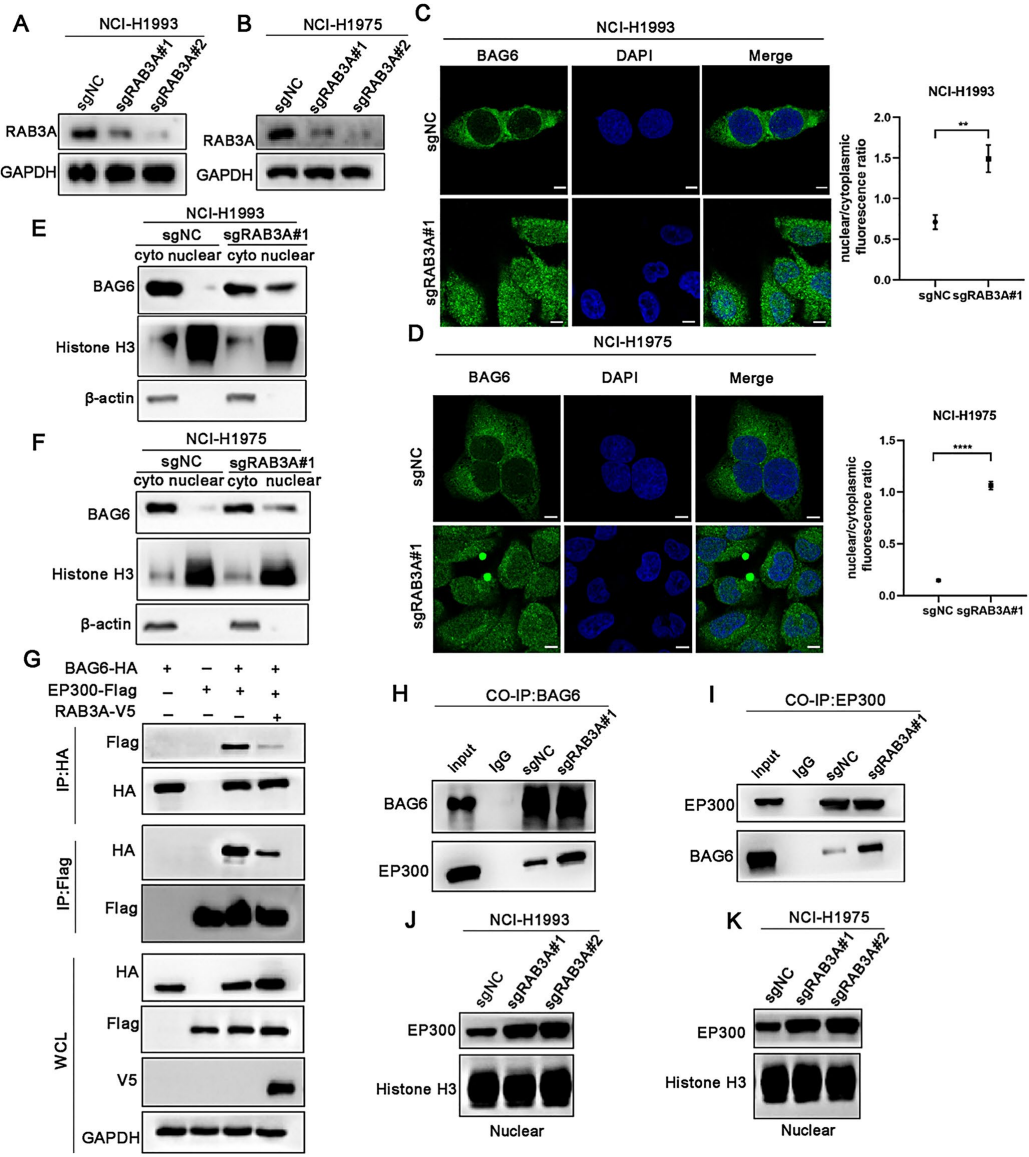
RAB3A depletion increases BAG6-EP300 nucleation. **A**,** B** Western blot of RAB3A-WT and RAB3A-knockdown cells to confirm RAB3A downregulation. **C**,** D** Immunofluorescence analysis of BAG6 localization in NCI-H1993 and NCI-H1975 cells with RAB3A knockdown. Scale bars: 5 μm. **E**,** F** Western blot analysis of cytosolic and nuclear fractions showing BAG6 levels in NCI-H1993 and NCI-H1975 cells with RAB3A knockdown. **G** Co-IP of BAG6 and EP300 in HEK-293T cells co-transfected with RAB3A-V5, EP300-Flag, or BAG6-HA plasmids. **H**,** I** Endogenous immunoprecipitation assay showing the interaction between BAG6 and EP300 in NCI-H1993 cells with RAB3A knockdown. IgG was used as a control. **J**,** K** Western blot analysis of nuclear EP300 levels in NCI-H1993 and NCI-H1975 cells with RAB3A knockdown. Error bars represent the mean ± SD. The significance level was represented by **p < 0.01, ****p < 0.0001

Increasing evidence suggests that RAB3A primarily serves a crucial function in regulating the recruitment, tethering, and docking of secretory vesicles to the plasma membrane [18]. To determine how RAB3A regulates the subcellular localization of BAG6, we transduced NCI-H1993 and NCI-H1975 cells with RAB3A targeting sgRNA and confirmed equivalent knockdown efficiency by western blot (Fig. 4A and B). The immunofluorescence assay showed that RAB3A knockdown induced BAG6 nuclear localization from cytosol as compared to the sgNC cells (Fig. 4C and D). Further cytosolic and nuclear extract fractionation assays manifested that RAB3A knockdown increased the nuclear levels of BAG6 (Fig. 4E and F). Recent studies have supported the translocation of EP300 into the nucleus is dependent on BAG6 [31–34], prompting us to investigate whether RAB3A could affect the interaction between BAG6 and EP300. We transfected RAB3A-V5, EP300-Flag or BAG6-HA plasmids into HEK-293T cells, respectively. We observed that the binding of EP300 to BAG6 was inhibited by RAB3A overexpression (Fig. 4G). Further coimmunoprecipitation assay showed knockdown RAB3A improved the binding of EP300 on BAG6 (Fig. 4H and I). Meanwhile, molecular docking revealed that the binding of RAB3A to BAG6 is energetically more favorable than that of EP300 to BAG6 providing a structural basis for the competitive binding that directs BAG6 translocation (Fig. S4). We further analyzed the EP300 levels of the nuclear. Consistently, RAB3A depletion increased nuclear EP300 levels (Fig. 4J and K). Taken together, these findings revealed that RAB3A depletion increased translocation of BAG6-EP300 in the nucleus.

### RAB3A knockdown increases the acetylation of p53 and Rb

**Fig. 5:**
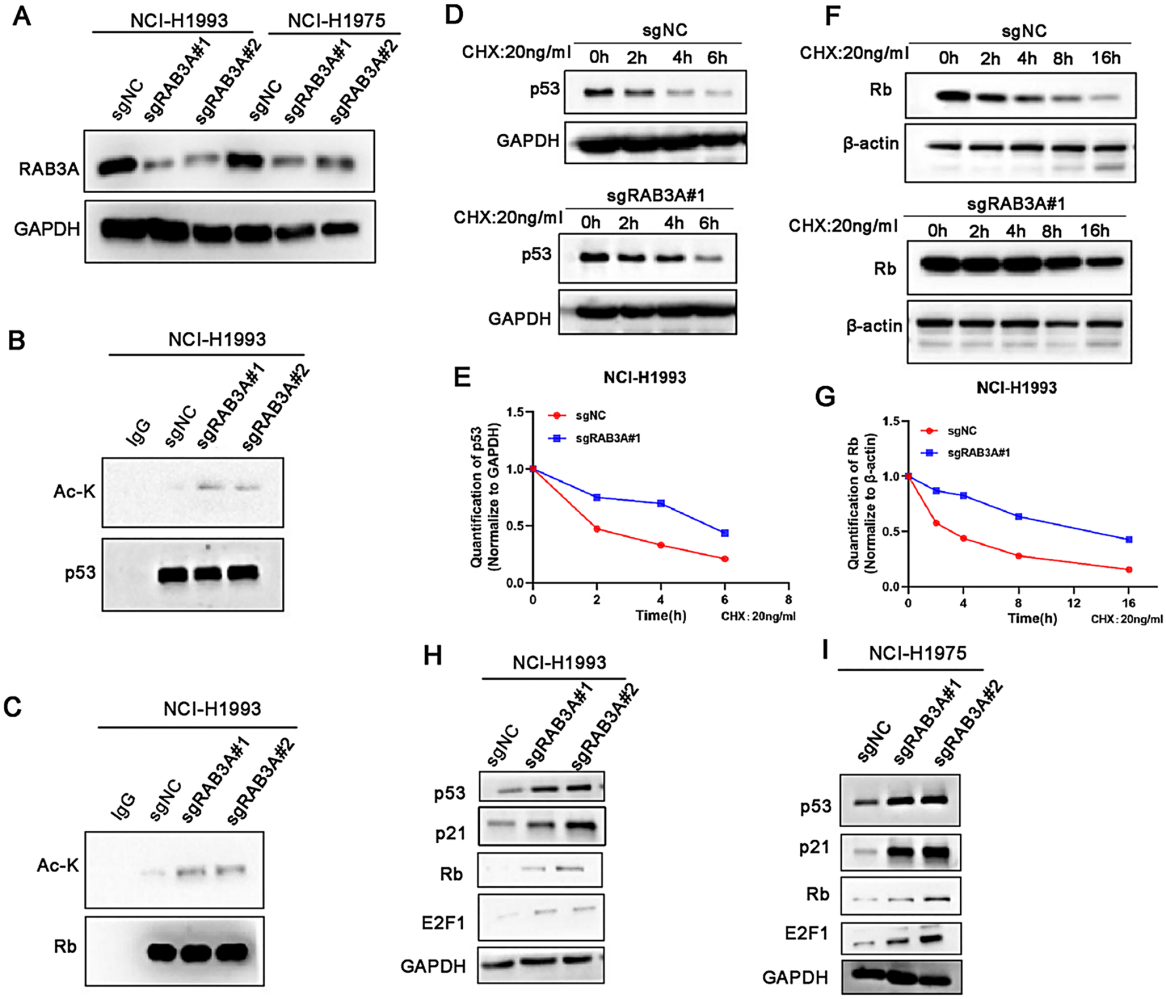
RAB3A knockdown enhances p53 and Rb stability. **A** Western blot of the indicated stable cells to confirm RAB3A downregulation. **B**,** C** Immunoprecipitation assay showing the acetylation levels of p53 and Rb. IgG was used as a control. **D**,** E** Western blot of p53 levels in the indicated stable NCI-H1993 cells treated with CHX (20 ng/ml). **F**,** G** Western blot of Rb levels in the indicated stable NCI-H1993 cells treated with CHX (20 ng/ml). **H**,** I** Western blot of p21 and E2F1 in the indicated stable cells

EP300 was reported to capable of acetylating p53 and Rb non-histone proteins [35, 36], we hypothesized that the increased nuclear levels of BAG6-EP300 mediated by RAB3A may increase the acetylation of the tumor suppressors p53 and Rb. To test the hypothesis, we modified NCI-H1993 and NCI-H1975 cell lines using RAB3A-targeting sgRNA. The equivalent knockdown efficiency was subsequently verified through western blot analysis (Fig. 5A). Subsequently, our data showed that RAB3A depletion increased the acetylation levels of p53 and Rb proteins (Fig. 5B and C). It was reported that the acetylation of p53 results in an extended half-life for endogenous p53 [37]. Meanwhile, Rb undergoes acetylation by E1A-recruited EP300, which maintains Rb in an active hypo-phosphorylation state [38]. To investigate the effect of acetylation on p53 and Rb proteins, we conducted cycloheximide (CHX) chase assay. The results showed that RAB3A knockdown extended the half-life of p53 and Rb (Fig. 5D-G). Subsequently, the downstream molecules of p53 and Rb were tested independently [39]. We observed that RAB3A knockdown upregulated p21 and E2F1 levels. (Fig. 5H and I). Overall, these results provide evidence that RAB3A knockdown increases the nuclear localization of BAG6-EP300, which further acetylates p53 and Rb proteins, thereby contributing to the suppression of NSCLC progression.

### RAB3A promotes BAG6-mediated mitophagy and attenuates the efficacy of cisplatin

**Fig. 6:**
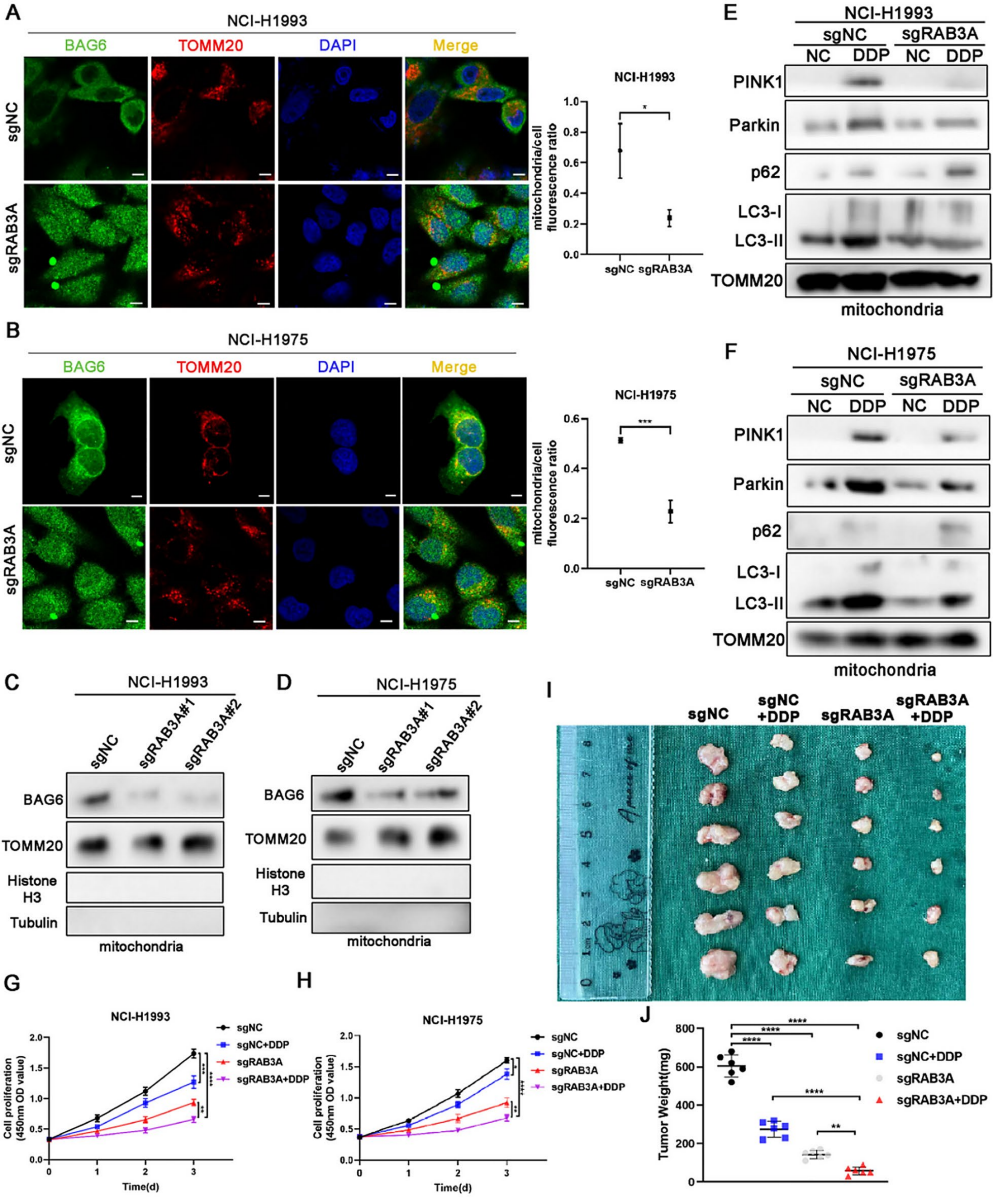
Mitophagy facilitated by RAB3A-induced BAG6 activation augments the therapeutic efficacy of cisplatin. **A**,** B** Immunofluorescence showing the localization of BAG6 in NCI-H1993 and NCI-H1975 cells with RAB3A knockdown. Scale bars: 5 μm. **C**,** D** Western blot of BAG6 expression after mitochondrial purification from NCI-H1993 and NCI-H1975 cells. **E**,** F** Relative protein expressions of PINK1, Parkin, p62, and LC3-II in NCI-H1993 and NCI-H1975 cells treated with DDP (10 µM). **G**,** H** CCK8 analysis of cell viability in NCI-H1993 and NCI-H1975 cells treated with DDP (10 µM). **I**,** J** Representative images of xenografts and tumor weight were shown. The animals were euthanized in 30 days (*n* = 6 mice/group). The significance level was represented by *p < 0.05, **p < 0.01, ****p < 0.0001. DDP, cisplatin

We next investigated whether RAB3A would regulate the mitochondrial localization of BAG6. Firstly, we performed an immunofluorescence assay to detect the localization of BAG6 in mitochondria. The results indicated that RAB3A depletion decreased the mitochondrial localization of BAG6 (Fig. 6A and B). To confirm mitochondrion-specific RAB3A-BAG6 interaction, Co-IP assays were performed using purified mitochondrial lysates. RAB3A co-immunoprecipitated BAG6 from mitochondrial fractions (Fig. S5). Similarly, mitochondrial purification from NCI-H1993 and NCI-H1975 cells, followed by western blot analysis, demonstrated that RAB3A knockdown reduced BAG6 levels in mitochondria (Fig. 6C and D). Considering the relationship between BAG6 and mitophagy, we selected cisplatin, a drug that could cause oxidative stress, to assess whether RAB3A could affect oxidative stress of NSCLC cells. Treatment of NCI-H1993 and NCI-H1975 cells with 10 µM cisplatin revealed that RAB3A knockdown reduced PINK1 and Parkin levels, while concurrently diminishing LC3-II and elevating p62, indicating suppressed mitophagic flux (Fig. 6E and F). Conversely, RAB3A overexpression augmented mitophagy, ultimately enhancing tumor cells’ tolerance to oxidative stress(Fig. S6). Given that mitophagy can counteract oxidative stress, we also examined the effect of RAB3A on cisplatin efficacy. The result showed that RAB3A knockdown enhances the ability of cisplatin to inhibit tumor cell proliferation(Fig. 6G and H). Consistently, the data of animal experiments showed that RAB3A knockdown improved the efficacy of cisplatin(Fig. 6I and J). Together these results suggest that RAB3A mediates the transport of BAG6 to mitochondria, thereby optimizing the therapeutic potential of cisplatin for non-small cell lung cancer treatment.

## Discussion

Non-small cell lung cancer (NSCLC) is an extremely dangerous disease, accounting for about 80% of lung cancer [40]. Here, we observed that RAB3A exhibits elevated expression in NSCLC tissues and correlates with poor survival. Furthermore, RAB3A knockdown inhibited the proliferation and clonogenic capacity of NSCLC cells. These observations indicate that RAB3A serves as a prognostic biomarker for NSCLC.

**Fig. 7:**
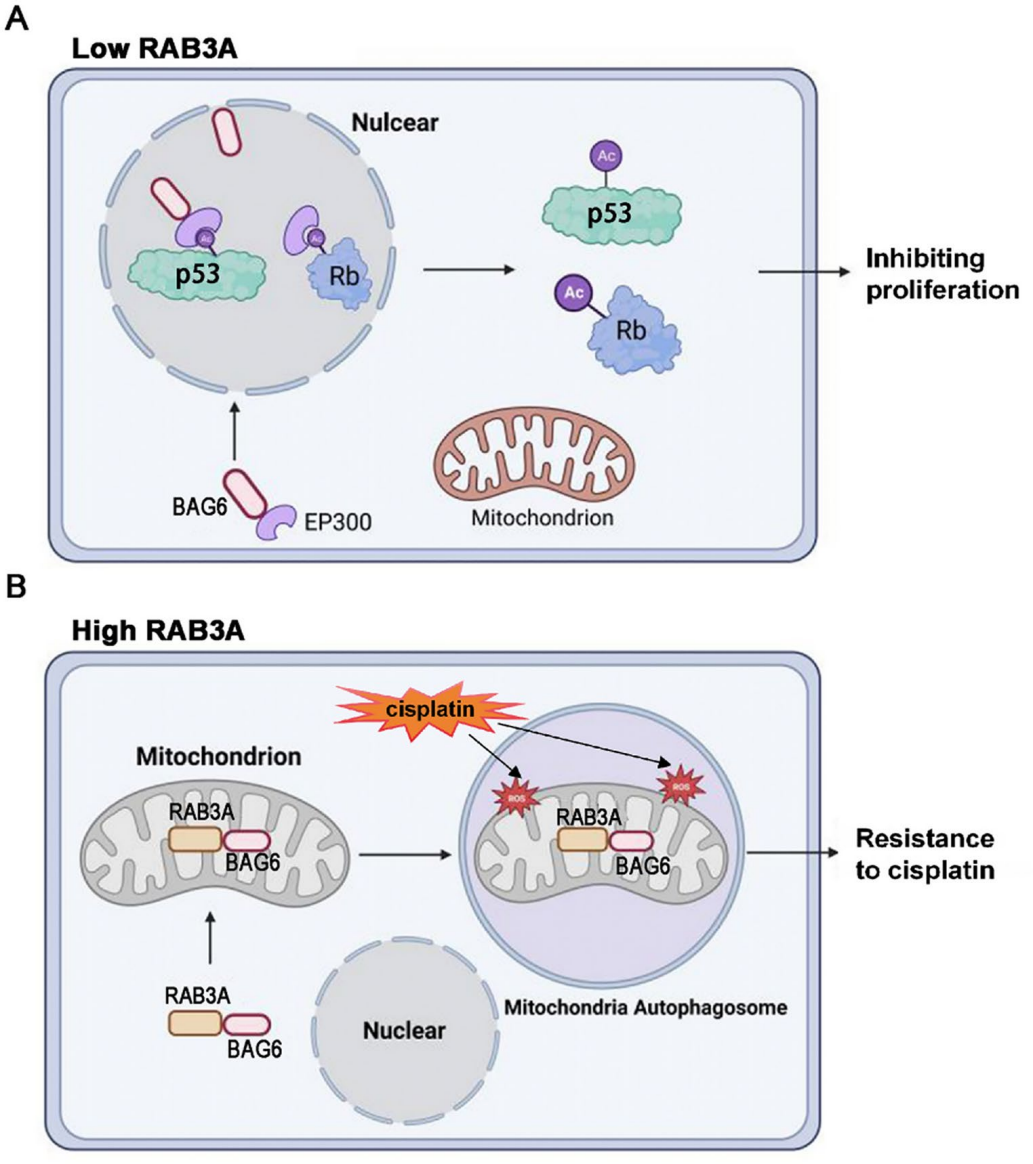
The schematic diagram describes the role of RAB3A in modulating BAG6 translocation in non-small cell lung cancer. **A** RAB3A knockdown facilitates the nuclear entry of BAG6-EP300 resulting in the increasing acetylation level of p53 and Rb, which inhibits the proliferation of NSCLC cells. **B** Amplification of RAB3A promotes the translocation of BAG6 to mitochondria to activate mitophagy, which enhances the resistance of NSCLC to cisplatin

Rabs are considered the main organizers of vesicle tethering [41]. Several studies demonstrate that RAB3A critically regulates mitochondrial bioenergetics, particularly mitochondrial oxidative metabolism [42–45]. Moreover, BAG6 functions as a central node in mitochondrial proteostasis, coupling mitophagy activation with degradation of metabolic regulators to enforce stress adaptation [30, 46–48]. Structurally, the UBL domain of BAG6 functions as a hydrophobic recognition module that binds client proteins bearing exposed hydrophobic residues [49]. RAB3A harbors critical hydrophobic interfacial residues indicating potential compatibility [50]. Notably, T. Takahashi et al. have confirmed that RAB3A were efficiently co-precipitated with BAG6 [51]. Although BAG6 ranked outside the top 10 MS candidates, it was prioritized for validation based on its known mitochondrial functions and domain compatibility with RAB3A. Crucially, Co-IP assays of the top 10 MS hits revealed no detectable interaction with RAB3A suggesting these represent nonspecific binders(Fig. S7). We performed immunoprecipitation experiments and verified that RAB3A and BAG6 interact with each other. However, the specific mechanism remains unclear. Notably, the downregulation or overexpression of RAB3A gene does not affect the total protein level of BAG6. Previous studies have revealed RAB3A participates in intracellular and extracellular transport [52–54]. As shown in immunofluorescence assay, BAG6 is mainly distributed in the cytoplasm of NSCLC cells. In this study, as illustrated in Fig. 7A, RAB3A depletion facilitates the nuclear entry of BAG6. Meanwhile, BAG6 carries EP300 into the nucleus, resulting in an increase in the acetylation level of p53 and Rb. The stability of p53 and Rb increases and activates downstream signal pathways, thus inhibiting the proliferation of NSCLC cells.

Accumulating evidence indicates that RAB3A regulates mitochondrial oxidative metabolism. Proteomic profiling of diabetic β-cells reveals synchronous upregulation of RAB3A and mitochondrial metabolic proteins [43]. In rosuvastatin-treated neurons, reduced RAB3A correlates with compromised mitochondrial bioenergetics, evidenced by diminished ATP production and attenuated ROS generation [42]. Mechanistically, RAB3A inhibition suppresses GLP-1-induced potentiation of glucose uptake, mitochondrial membrane potential, and ATP synthesis in β-cells [44]. Collectively, these findings establish RAB3A as a critical modulator of mitochondrial oxidative metabolism. Functionally complementary, BAG6 is a nucleocytoplasmic shuttling protein that orchestrates mitophagy initiation, critically modulates PINK1 stability, and participates in the ubiquitin-mediated degradation of key regulators of mitochondrial ATP production, thereby playing a pivotal role in maintaining mitochondrial homeostasis under stress [30, 46–48]. In this study, based on the joint role of RAB3A and BAG6 in mitochondrial oxidative metabolism, we found that RAB3A transports BAG6 into mitochondria. Furthermore, mitochondrial purification revealed that RAB3A depletion under oxidative stress decreased levels of key mitophagy regulators in mitochondria.

Autophagy is a cellular catabolic pathway involving the degradation and recycling of proteins and organelles between isolated vesicles, autophagosomes, and lysosomes that provide hydrolases [55]. Autophagy plays two opposite roles at different stages of tumorigenesis [56, 57]. In tumors, autophagy inhibits tumors by limiting oxidative stress and inhibiting genomic instability of carcinogenic mutations [58]. However, autophagy also enables tumor cells to resist drugs, thereby greatly enhancing their ability to survive in complex and harsh environments [59]. Selective autophagy facilitates the degradation of compromised or depolarized mitochondria through a process known as mitophagy [60]. Here, we found that RAB3A knockdown made cells sensitive to cisplatin. Conversely, the presence of RAB3A strengthened the resistance of NSCLC to cisplatin. Interestingly, compelling evidence suggests that cisplatin accumulates within the mitochondrial matrix, thereby disrupting mitochondrial respiration and contributing to oxidative stress [61]. Meanwhile, mitophagy mediates cell resistance to oxidative stress, and BAG6 has been reported to be the inducer of mitophagy [30, 62]. Another study showed that BAG6 contains many domains, including nuclear output signal and nuclear location signal [32]. According to these data, when NSCLC cells experience oxidative stress, the high expression of RAB3A mediates the transportation of BAG6 to mitochondria. Thereby, BAG6-mediated mitophagy enables NSCLC cells to better resist oxidative stress, thereby promoting NSCLC progression (Fig. 7B). However, the motif of RAB3A-BAG6 interaction is still unclear. We will explore the functional domain of the interaction between RAB3A and BAG6 in future studies.

In summary, our data reveal that RAB3A expression is elevated in patients with NSCLC, exhibiting a correlation with poor prognosis. Further results indicate that RAB3A knockdown promotes BAG6-EP300 into the nucleus, increasing the stability of tumor suppressor genes and inhibiting tumor proliferation. Meanwhile, RAB3A facilitates the mitochondrial translocation of BAG6, thereby inducing mitophagy and diminishing the efficacy of cisplatin. Therefore, this work provides a basis for designing potential therapeutic approaches for NSCLC patients.

## Supplementary Information

Below is the link to the electronic supplementary material.

Supplementary Material 1

Supplementary Material 2

Supplementary Material 3

Supplementary Material 4

Supplementary Material 5

## Data Availability

The data that support the findings of this study are available on request from the corresponding author upon reasonable request.
