# Supplementary material for: RAB3A-mediated BAG6 translocation promotes non-small cell lung cancer tumorigenesis and progression
Source: Cell Oncol (Dordr). 2025 Oct 22;48(6):2001–15. doi: 10.1007/s13402-025-01123-z (PMC12698754; doi:10.1007/s13402-025-01123-z)
Supplement: Supplementary file 2 — Supplementary Material 2 [file 13402_2025_1123_MOESM2_ESM.docx]

**RAB3A-mediated BAG6 translocation promotes non-small cell lung cancer tumorigenesis and progression**

Xiaoli Liu^1,2^, Wen Wang^1^, Wanwei Cao^3^, Zhanyu Li^3^, Hao Huang^3^, Fang Liu ^4^, Yang Wang ^4^, Zhijuan Zhong ^4^, Hongyu Zhang^1^, Xiaofeng Pei^1^, Hongtao Chen^4,5^

^1^Department of Oncology, The Fifth Affiliated Hospital of Sun Yat-sen University, Zhuhai 519000, China.

^2^Guangdong Provincial Engineering Research Center of Molecular Imaging, the Fifth Affiliated Hospital of Sun Yat-sen University, Zhuhai 519000, China

^3^Department of Pathology, The Fifth Affiliated Hospital of Sun Yat-sen University, Zhuhai 519000, China.

^4^Department of Laboratory, The Fifth Affiliated Hospital of Sun Yat-sen University, Zhuhai 519000, China.

^5^Department of Laboratory, Xinjiang Medical University Affiliated Second Hospital, Wulumuqi 830028, China.

Xiaoli Liu, Wen Wang, Wanwei Cao and Zhanyu Li contributed equally.

**Email:**

Xiaoli Liu:[liuxli37@mail2.sysu.edu.cn](mailto:liuxli37@mail2.sysu.edu.cn)

Wen Wang: wangw258@mail3.sysu.edu.cn

Wanwei Cao: caoww@mail.sysu.edu.cn

Zhanyu Li: lizhany@mail.sysu.edu.cn

Hao Huang: [huangh227@outlook.com](mailto:huangh227@outlook.com)

Fang Liu: liufang17961051@outlook.com

Yang Wang: 13639908783@139.com

Zhijuan Zhong: 13570633297@139.com

Hongyu Zhang: [zhhyu@mail.sysu.edu.cn](mailto:zhhyu@mail.sysu.edu.cn)

Xiaofeng Pei: [peixf3@mail.sysu.edu.cn](mailto:peixf3@mail.sysu.edu.cn)

Hongtao Chen: [13926925941@139.com](mailto:13926925941@139.com)

**Corresponding author at:**

**Hongyu Zhang**

Department of Oncology, The Fifth Affiliated Hospital of Sun Yat-sen University, Zhuhai 519000, China

Email: [zhhyu@mail.sysu.edu.cn](mailto:zhhyu@mail.sysu.edu.cn)

**Xiaofeng Pei**

Department of Oncology, The Fifth Affiliated Hospital of Sun Yat-sen University, Zhuhai 519000, China

Email: peixf3@mail.sysu.edu.cn

**Hongtao Chen**

Department of Laboratory, Xinjiang Medical University Affiliated Second Hospital, Wulumuqi 830028, China

Email: [13926925941@139.com](mailto:13926925941@139.com)

**Supplementary Materials**

Supplementary materials contain 8 supplementary figures.

**Supplementary Figures and Legends**

###
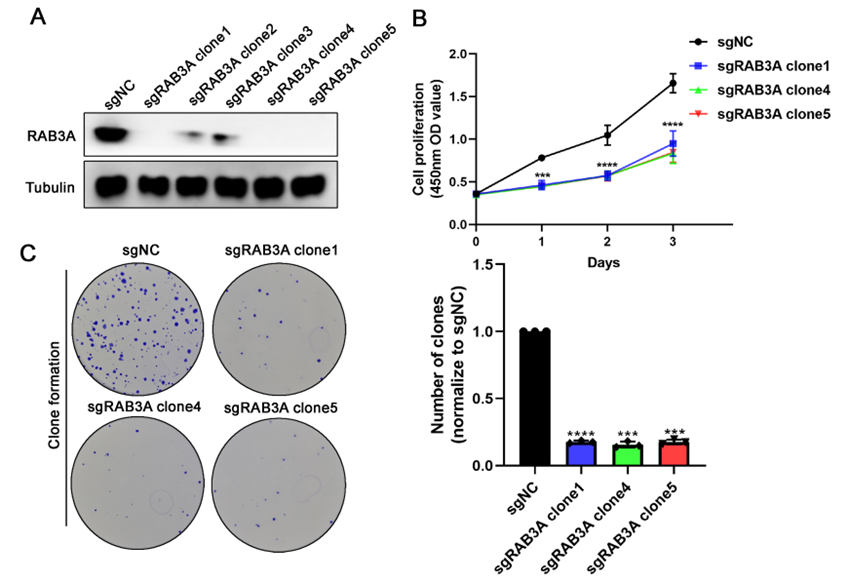


**Fig. S1** Phenotypic validation with RAB3A-null monoclonal lines.

**A** Western blot validation of RAB3A-null clones. **B** CCK8 assay of cell viability in RAB3A-null clones. **C** Colony formation assay in RAB3A-null clones. Data are presented as the mean ± SD. *** *p* < 0.001, **** *p* < 0.0001.

###
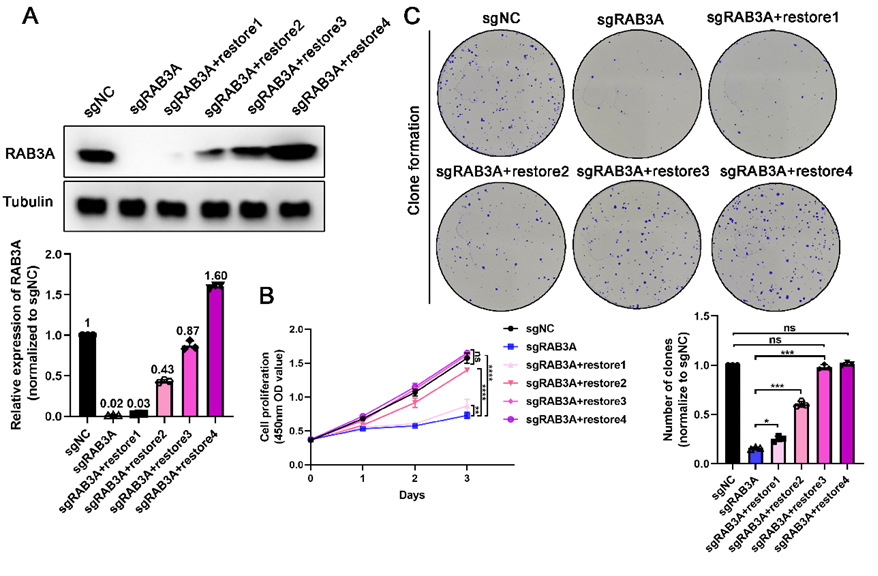


**Fig. S2** Physiological RAB3A levels suffice for oncogenic function.

**A** Experimental design for RAB3A titration. Lane 1: sgNC; Lane 2: sgRAB3A; Lanes 3-6: Rescue with very low/low/medium/high viral titers. **B** Proliferation in reconstituted cells. **C** Colony formation in reconstituted cells. * *p*<0.05, ** *p*<0.01,*** *p*<0.001, **** *p*<0.0001. ns, nonsignificant difference

###

**Fig. S3** RAB3A expression-dependent BAG6 cytoplasmic co-localization in NSCLC clinical specimens.

**A** Dual-IHC analysis of RAB3A (brown) and BAG6 (red) in RAB3A-low (left) and RAB3A-high (right) specimens . Scale bars: 625 μm (4×), 100 μm (20×).

###
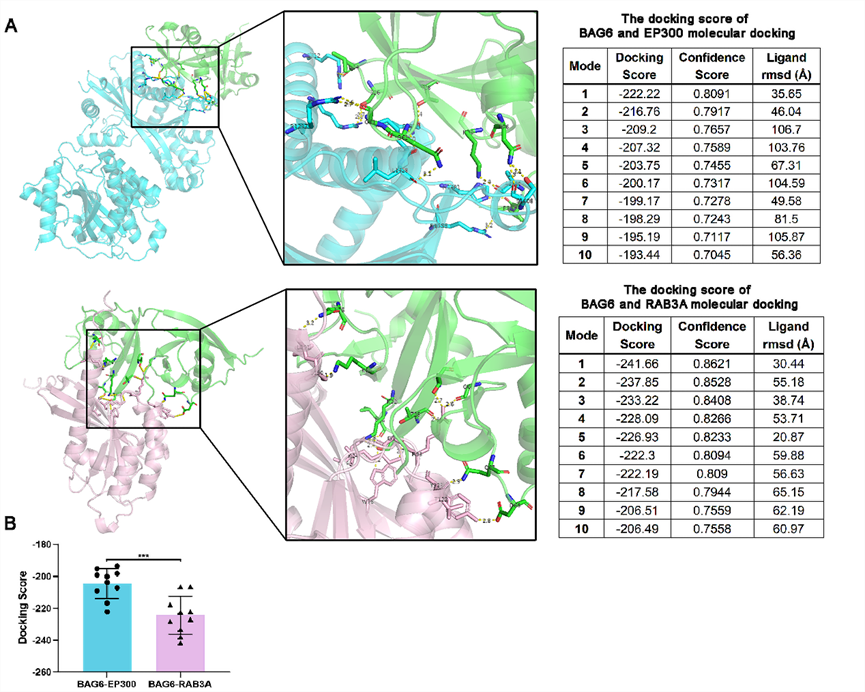


**Fig. S4** Computational modeling of competitive binding between RAB3A and EP300 for BAG6 recruitment.

**A** Molecular docking of protein complexes using HDOCK server. **B** Statistical analysis of HDOCK docking scores for protein complexes. Lower scores indicate higher affinity. Error bars represent the mean ± SD. ****p* < 0.001.


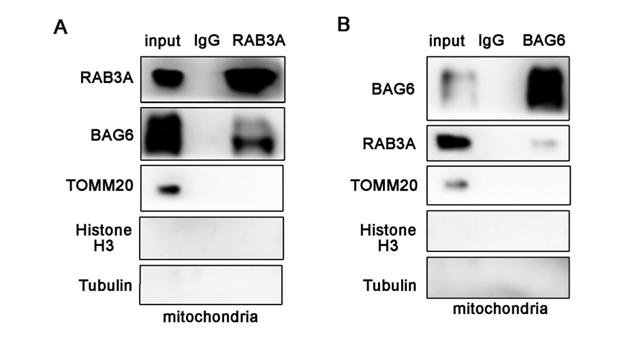


**Fig. S5** Co-IP of RAB3A and BAG6 from purified mitochondrial lysates.


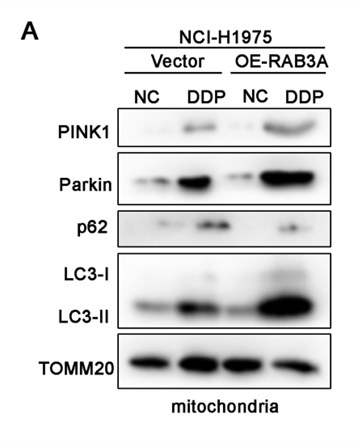


**Fig. S****6** RAB3A overexpression enhances mitophagy to confer oxidative stress tolerance.

**A** Western blot analysis of mitophagy markers in RAB3A overexpression cells treated with DDP (10 μM). DDP, cisplatin

###
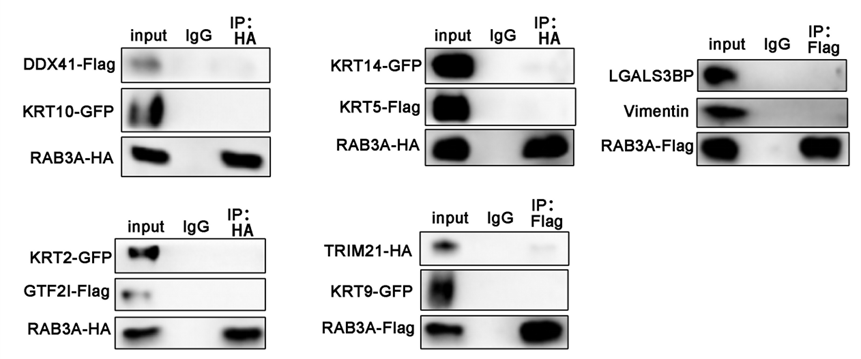


**Fig. S7** Validation of top 10 MS candidates.


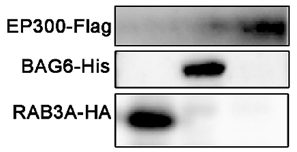


**Fig. S8** Western blot analysis of purified proteins.
