# Supplementary material for: RAB3A-mediated BAG6 translocation promotes non-small cell lung cancer tumorigenesis and progression
Source: Cell Oncol (Dordr). 2025 Oct 22;48(6):2001–15. doi: 10.1007/s13402-025-01123-z (PMC12698754; doi:10.1007/s13402-025-01123-z)
Supplement: Supplementary file 3 — Supplementary Material 3 [file 13402_2025_1123_MOESM3_ESM.docx]

**Supplementary Material 1 Supplementary methods**

**1 Methods**

**1.1 Molecular docking**

The BAG6 structure (PDB ID:4DWF; PDB DOI: <https://doi.org/10.2210/pdb4DWF/pdb>) and EP300 structure (PDB ID:6PGU) were retrieved from the Protein Data Bank[1-3]. The three-dimensional structure of human RAB3A (UniProt ID: P20336) was predicted using AlphaFold2(version 2.3.0) with default parameters[4, 5]. Protein-protein docking was performed using HDOCK server(<http://hdock.phys.hust.edu.cn/>) [6-10], utilizing residues within 5Å of the reported functional site. Top-ranked complexes were visualized in PyMOL v2.5.4[11].

**1.2 Double Immunohistochemistry**

Paraffin-embedded sections were baked at 65°C for 1 hour, followed by dewaxing. Heat-induced epitope retrieval was performed in citrate buffer (pH 6.0) at 100°C for 32 minutes. Sections were incubated with 100 µL of rabbit anti-RAB3A primary antibody (1:100 dilution; Proteintech, Inc., USA) at 37°C for 24 min. Brown chromogen was produceed using the OptiView DAB IHC Detection Kit (760-700), according to the manufacturer's instructions. Additionally, the sections were incubated with 100 µL of rabbit anti-BAG6 primary antibody (1:200 dilution; Proteintech Group, Inc., USA) at 37°C for 24 min. Red chromogen was resulted in using the UltraView Universal Alkaline Phosphatase Red Detection Kit (760-501), according to the manufacturer's instructions. The sections were counterstained with hematoxylin, dehydrated, cleared, and mounted using a synthetic resin mounting medium. Slides were digitally scanned using a Digital Pathology Slide Scanner(KF-PRO-005-EX; KFBIO).

### References

1. H. M. Berman *et al.*, The Protein Data Bank. *Nucleic acids research* **28**, 235-242 (2000).

2. A. S. Gardberg *et al.*, Make the right measurement: Discovery of an allosteric inhibition site for p300-HAT. *Structural dynamics (Melville, N.Y.)* **6**, 054702 (2019).

3. Stephen K. Burley *et al.*, Updated resources for exploring experimentally-determined PDB structures and Computed Structure Models at the RCSB Protein Data Bank. *Nucleic acids research* **53**, D564-D574 (2024).

4. J. Jumper *et al.*, Highly accurate protein structure prediction with AlphaFold. *Nature* **596**, 583-589 (2021).

5. M. Varadi *et al.*, AlphaFold Protein Structure Database in 2024: providing structure coverage for over 214 million protein sequences. *Nucleic acids research* **52**, D368-d375 (2024).

6. Y. Yan, H. Tao, J. He, S. Y. Huang, The HDOCK server for integrated protein-protein docking. *Nature protocols* **15**, 1829-1852 (2020).

7. Y. Yan, D. Zhang, P. Zhou, B. Li, S. Y. Huang, HDOCK: a web server for protein-protein and protein-DNA/RNA docking based on a hybrid strategy. *Nucleic acids research* **45**, W365-w373 (2017).

8. Y. Yan, Z. Wen, X. Wang, S. Y. Huang, Addressing recent docking challenges: A hybrid strategy to integrate template-based and free protein-protein docking. *Proteins* **85**, 497-512 (2017).

9. S. Y. Huang, X. Zou, A knowledge-based scoring function for protein-RNA interactions derived from a statistical mechanics-based iterative method. *Nucleic acids research* **42**, e55 (2014).

10. S. Y. Huang, X. Zou, An iterative knowledge-based scoring function for protein-protein recognition. *Proteins* **72**, 557-579 (2008).

11. L. L. C. Schrodinger, The PyMOL Molecular Graphics System, Version 2.0. . [**https://pymol.org/2/**](https://pymol.org/2/), (2017).
