# Supplementary material for: RAB3A-mediated BAG6 translocation promotes non-small cell lung cancer tumorigenesis and progression
Source: Cell Oncol (Dordr). 2025 Oct 22;48(6):2001–15. doi: 10.1007/s13402-025-01123-z (PMC12698754; doi:10.1007/s13402-025-01123-z)

Fig. 2A and H

Raw data

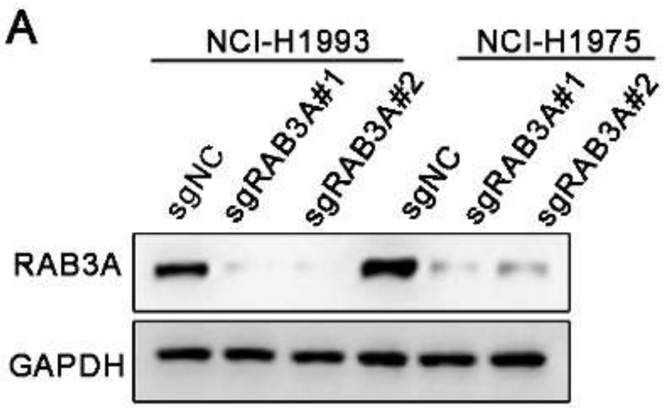

Fig. 2A

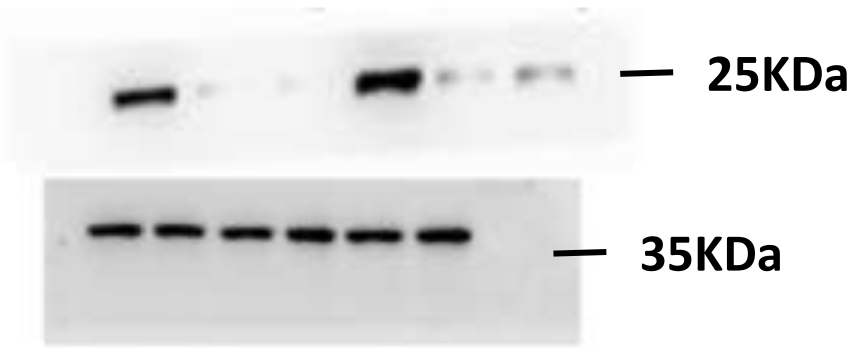

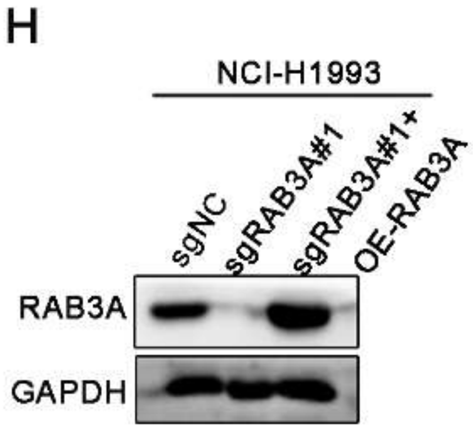

Fig. 2H

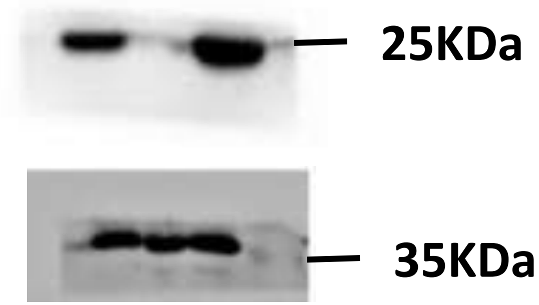

Fig. 3B

Raw data

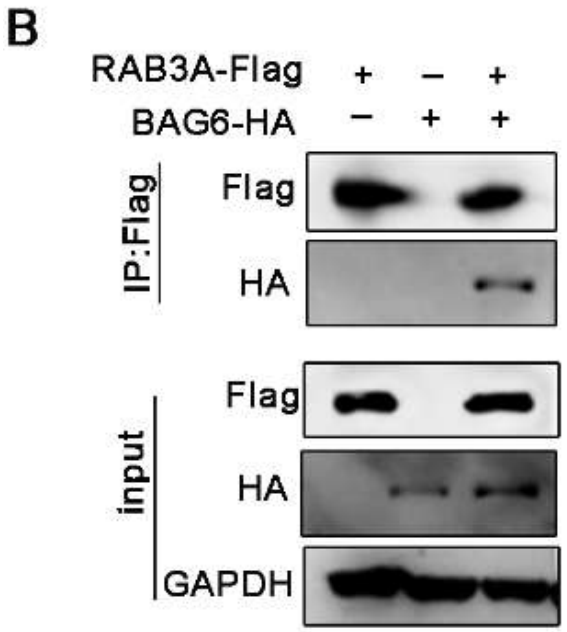

Fig. 3B

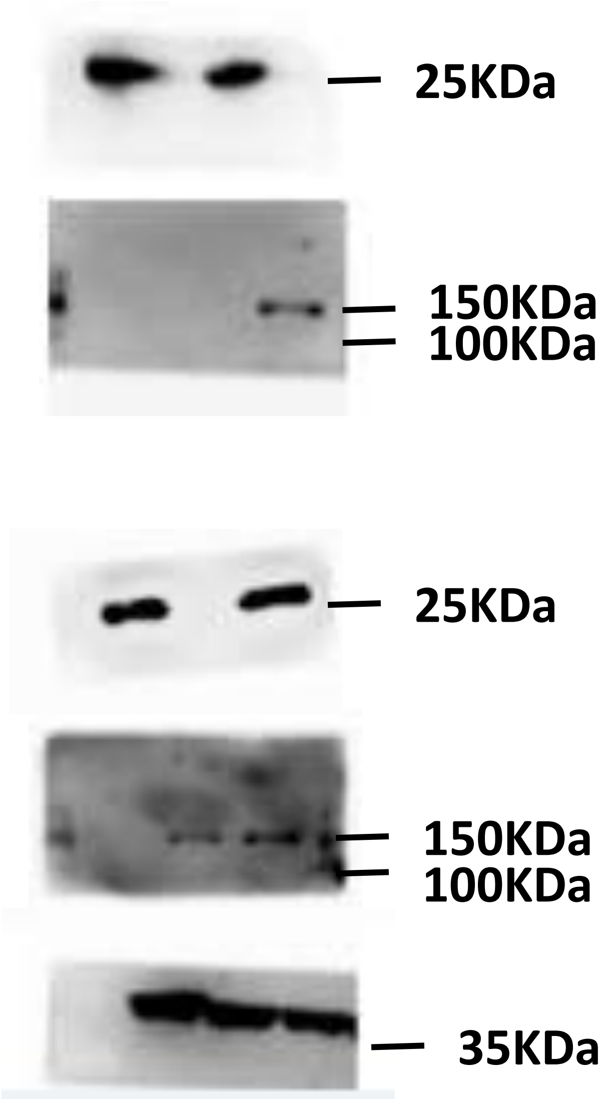

Fig. 3C

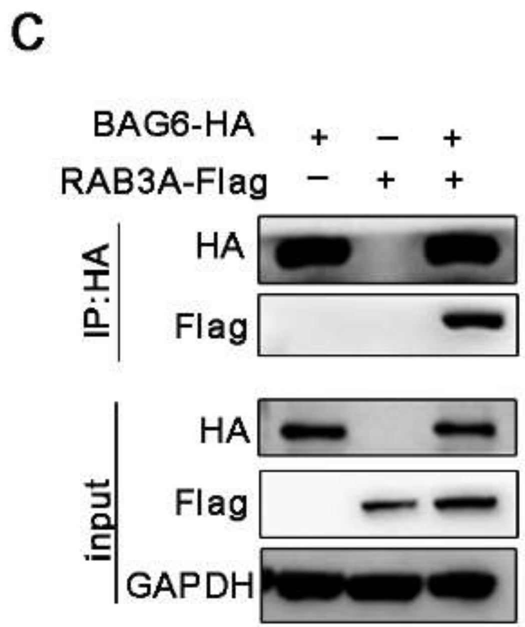

Raw data

Fig. 3C

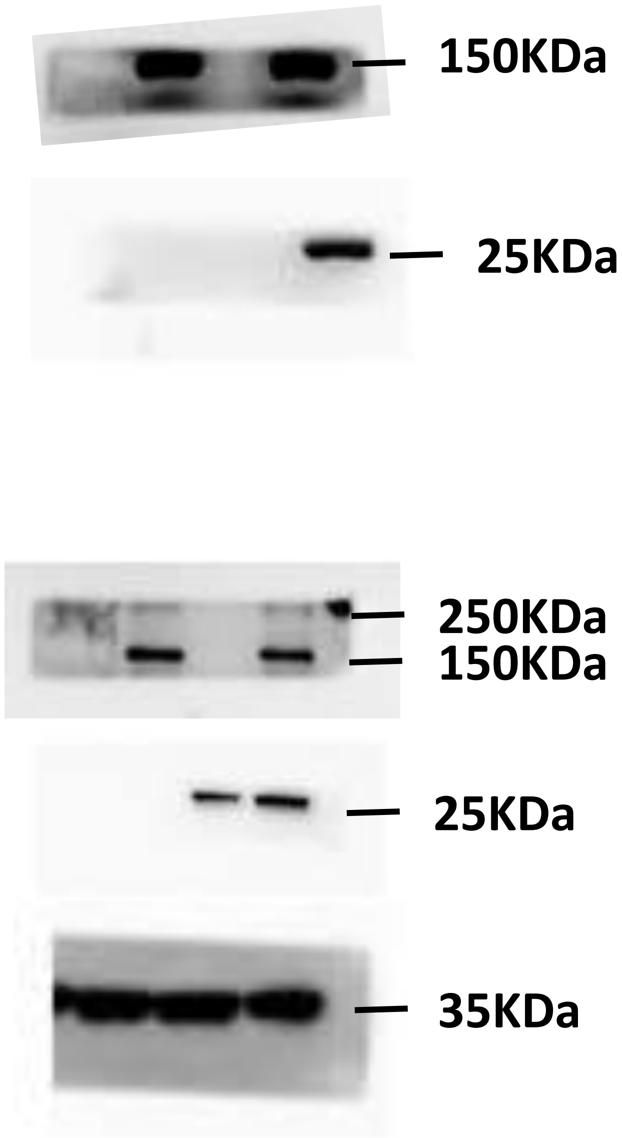

Fig. 3D-E

Raw data

D

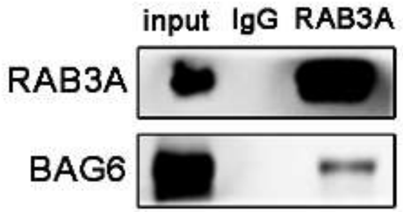

E

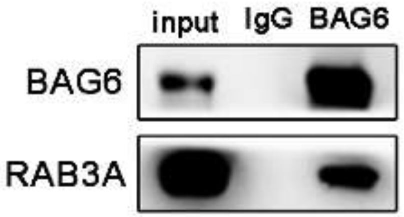

Fig. 3D

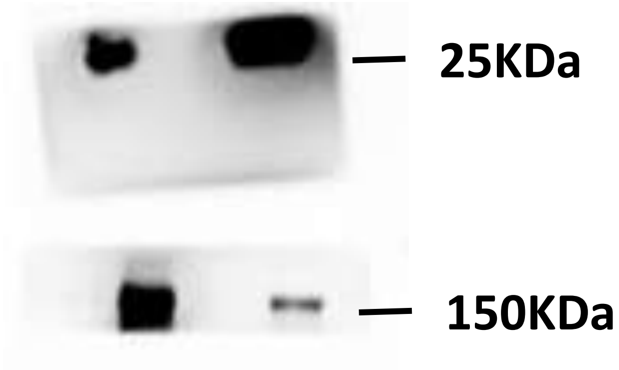

Fig. 3E

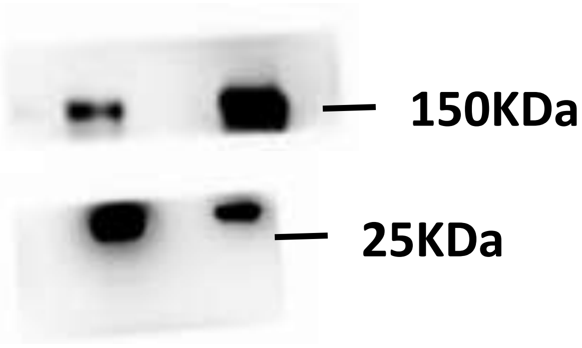

Fig. 3F-G

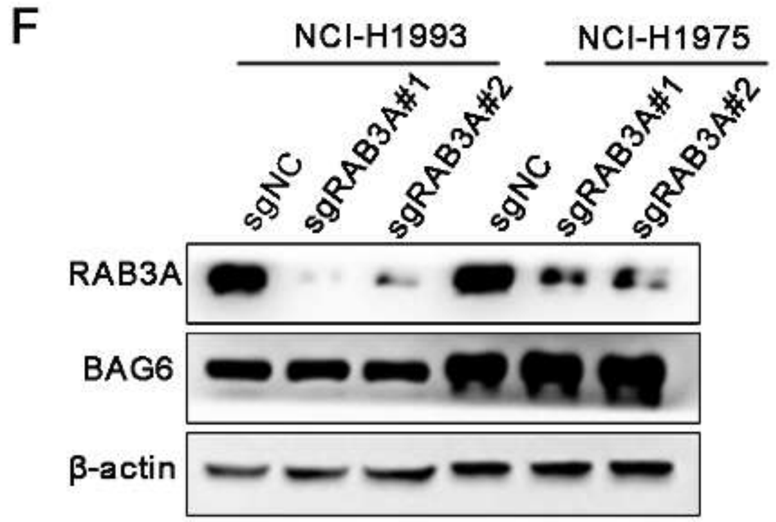

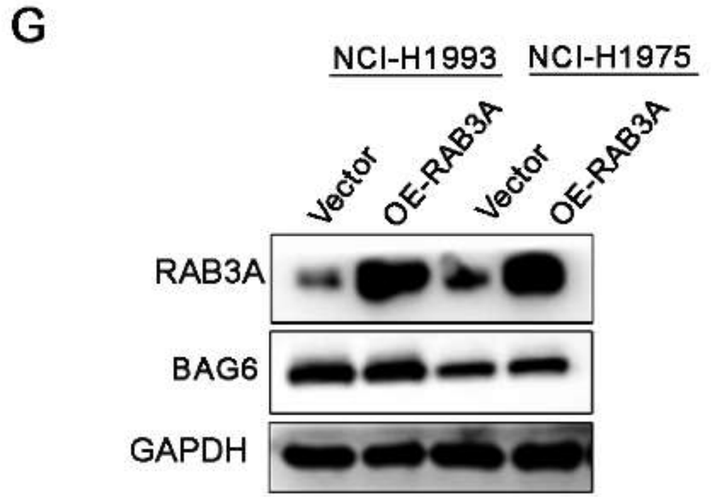

Raw data

Fig. 3F

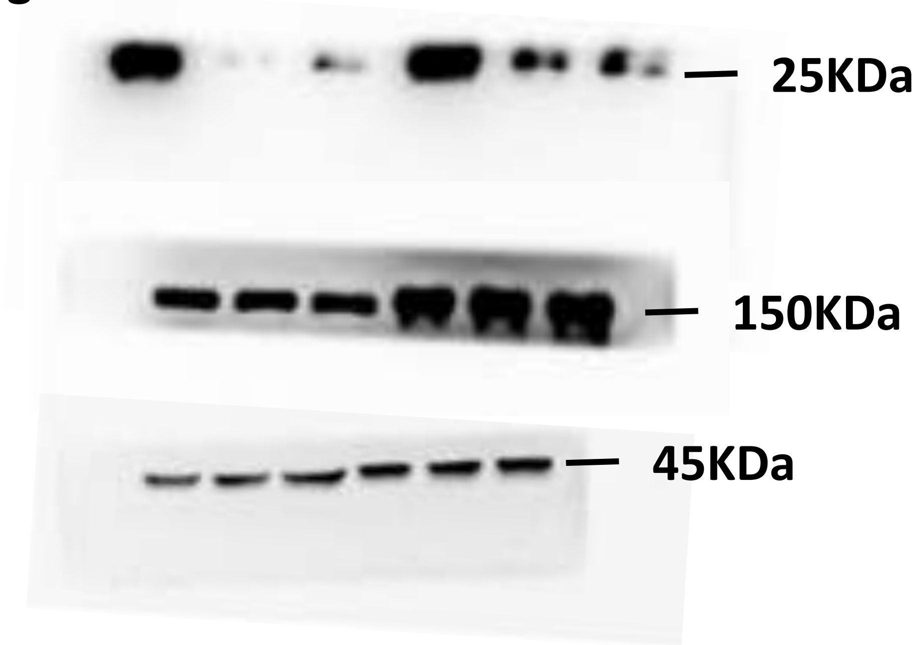

Fig. 3G

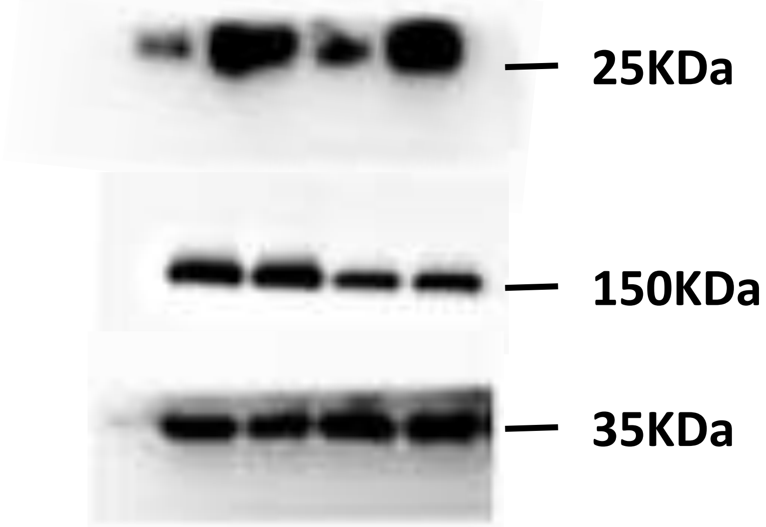

Fig. 4A-B

Raw data

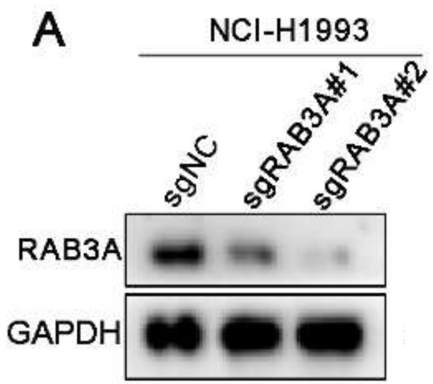

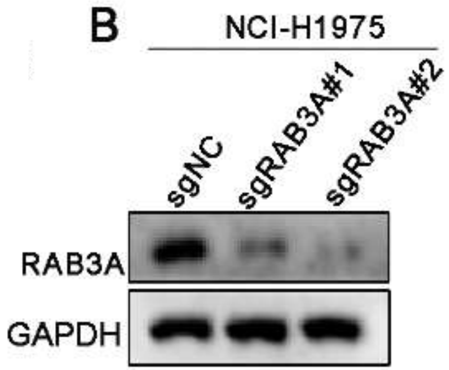

Fig. 4A

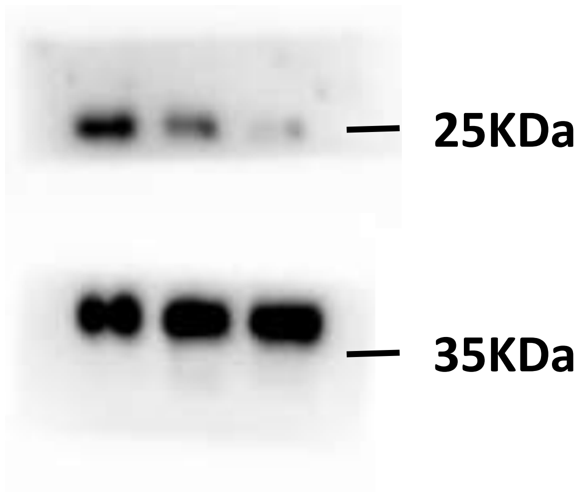

Fig. 4B

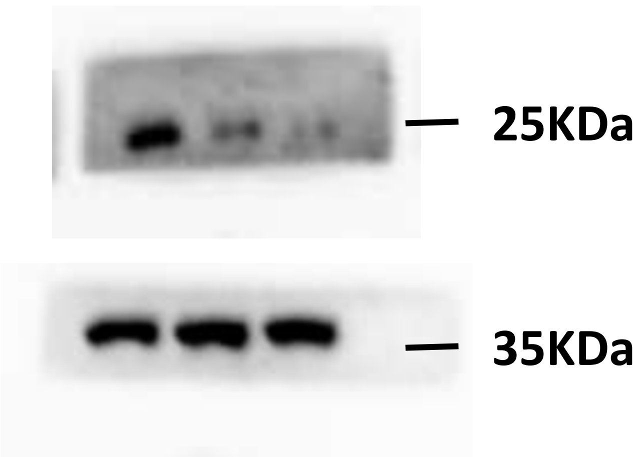

Fig. 4E-F

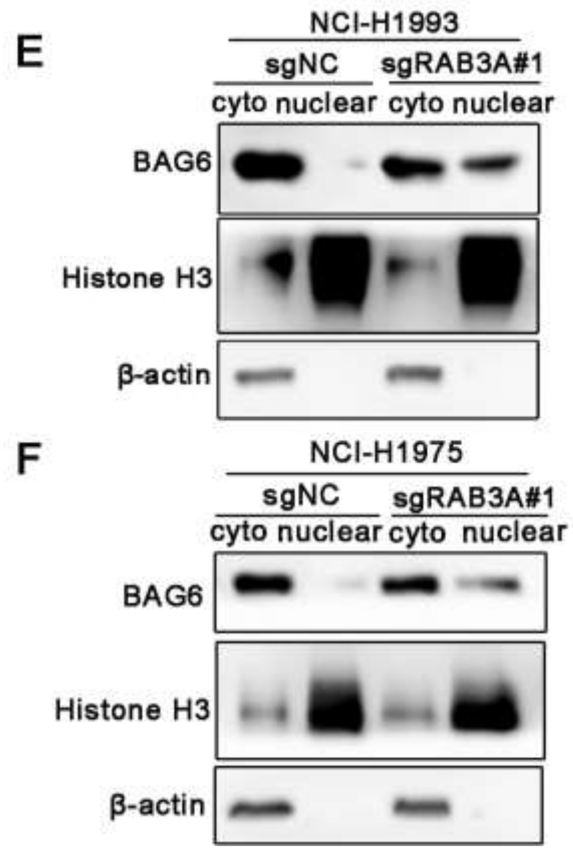

Raw data

Fig. 4E

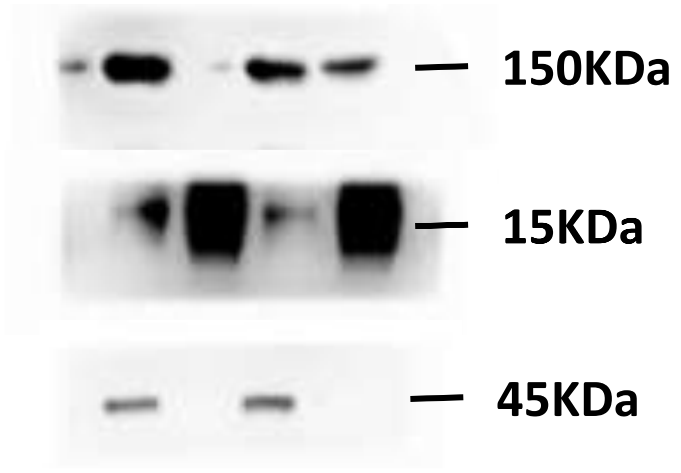

Fig. 4F

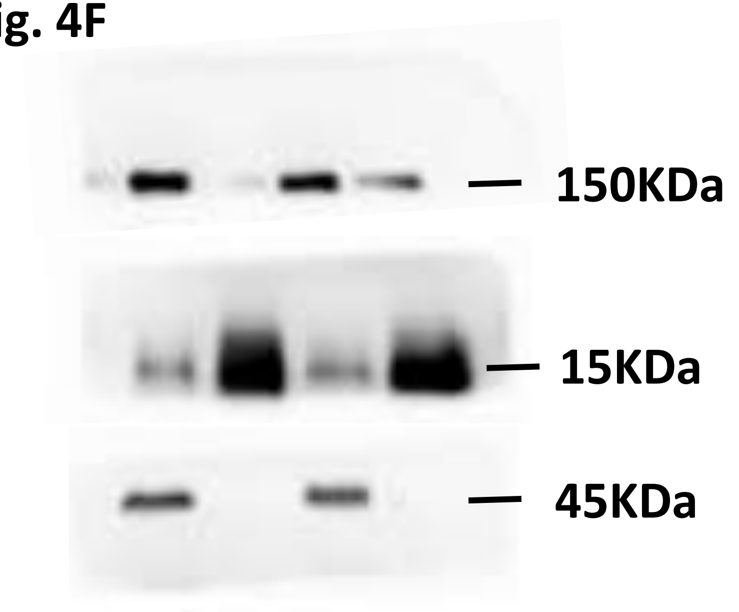

Fig. 4G

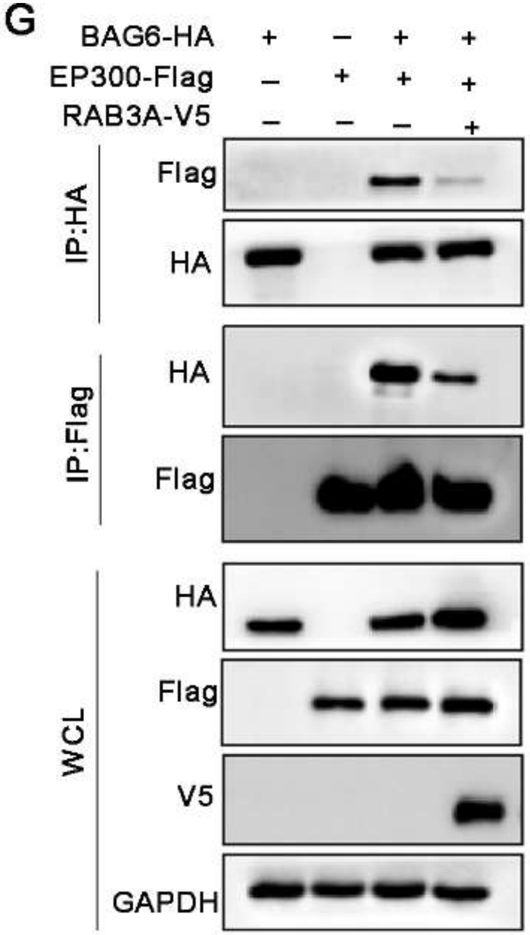

Raw data

Fig. 4G

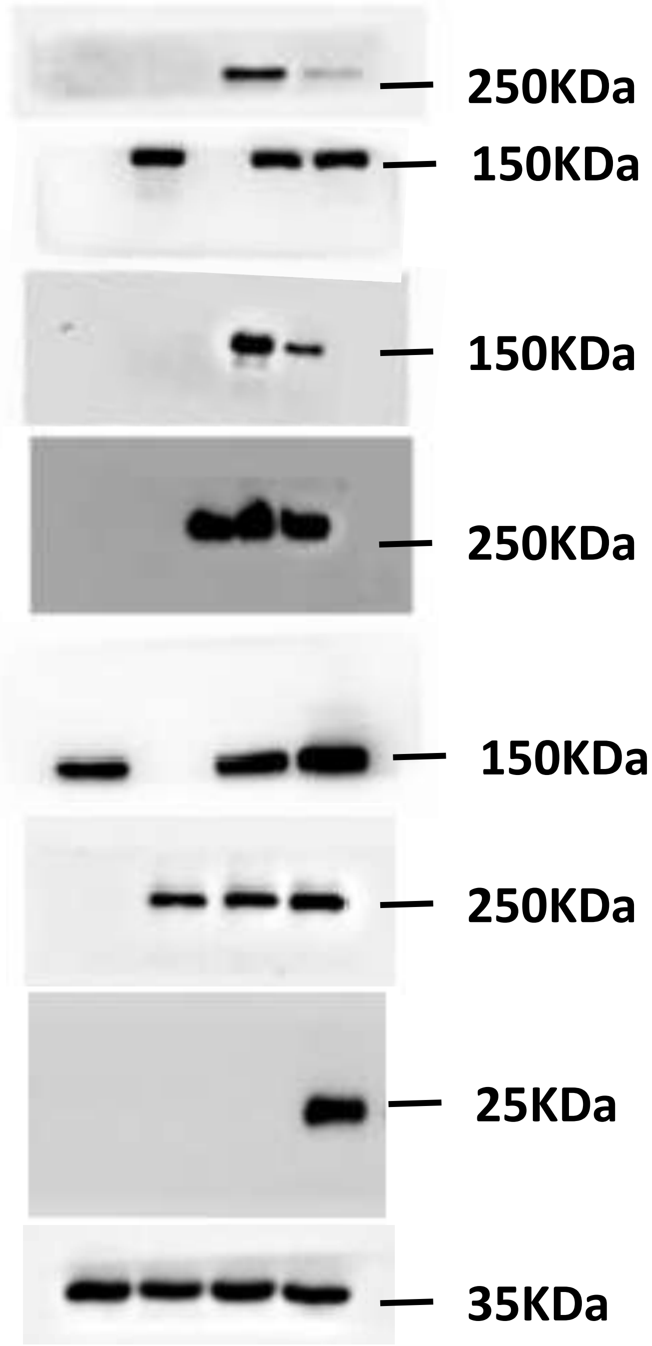

Fig. 4 H-I

Raw data

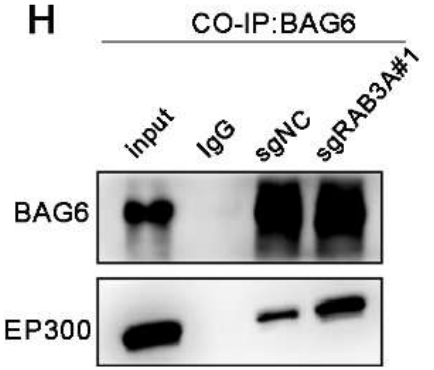

Fig. 4H

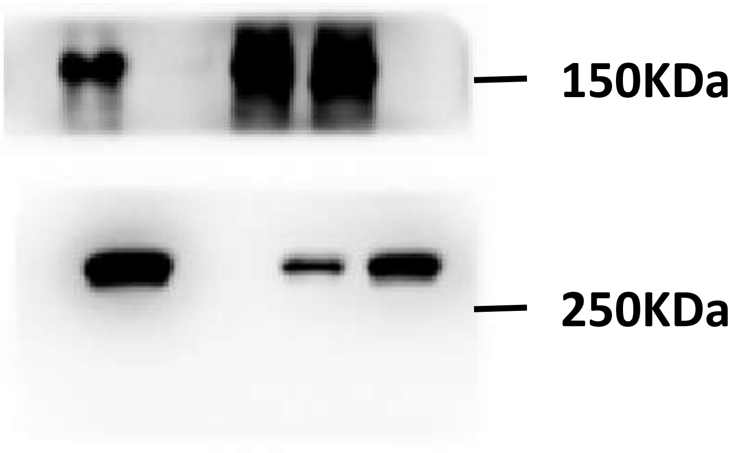

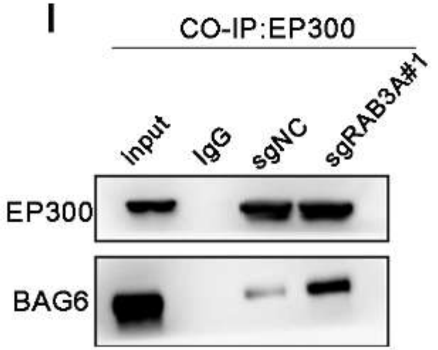

Fig. 4I

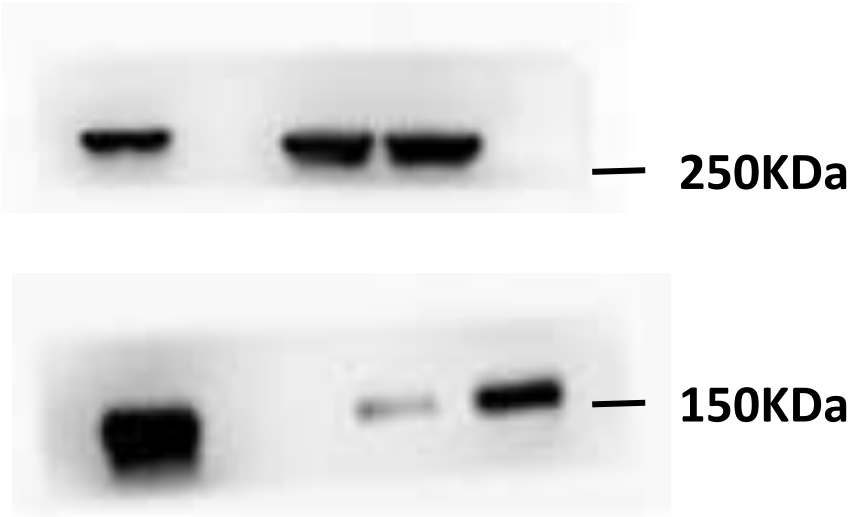

Fig. 4 J-K

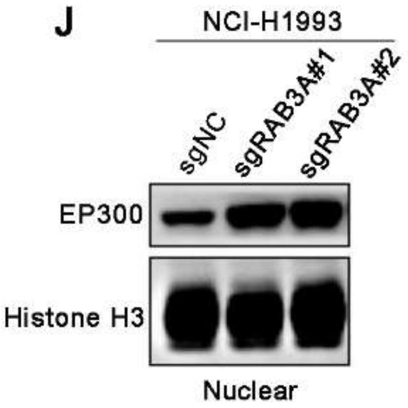

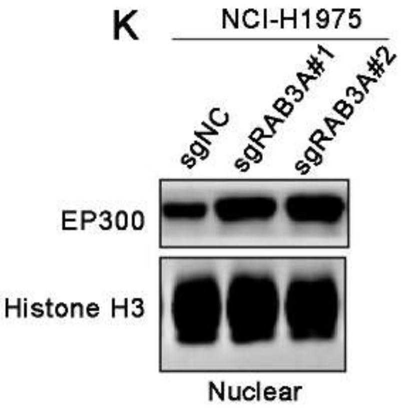

Raw data

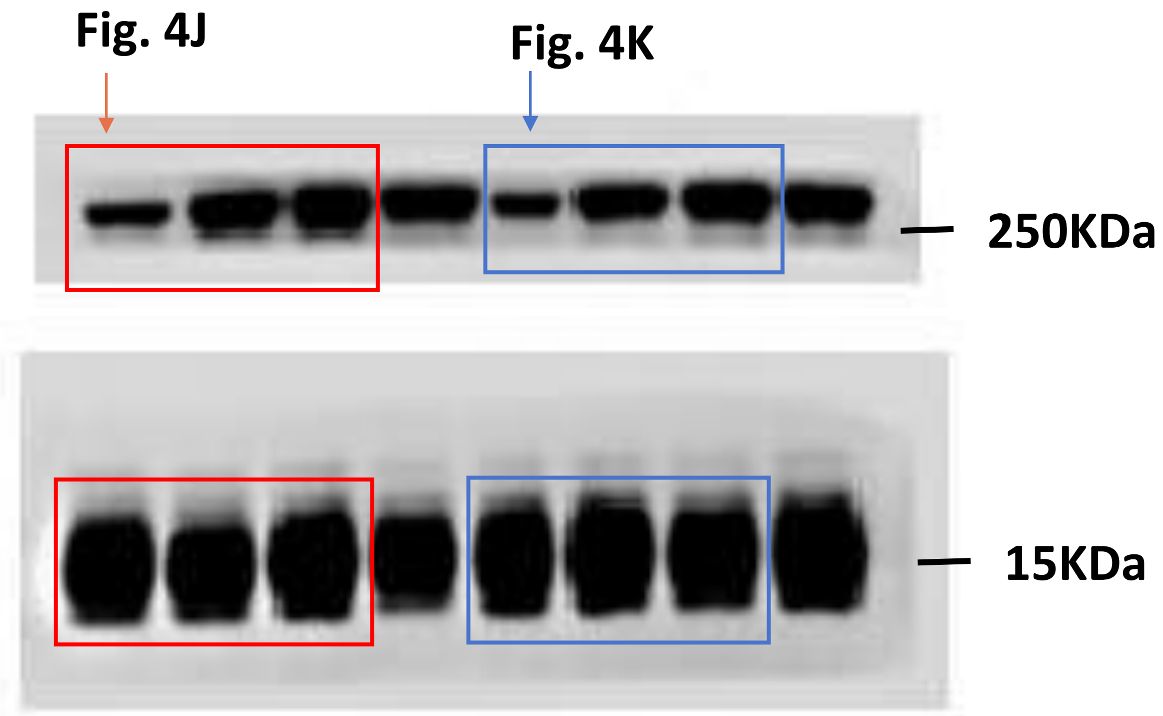

Fig. 5A

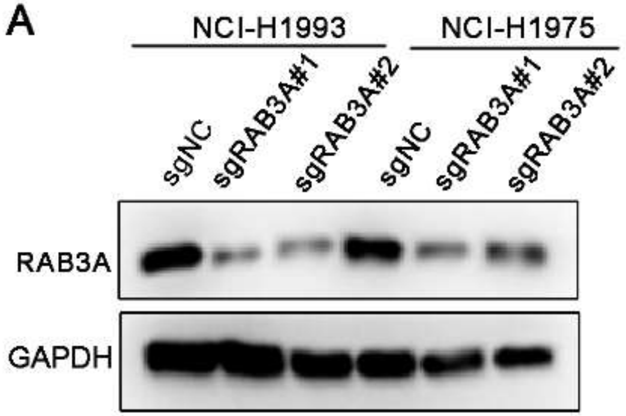

Raw data

Fig. 5A

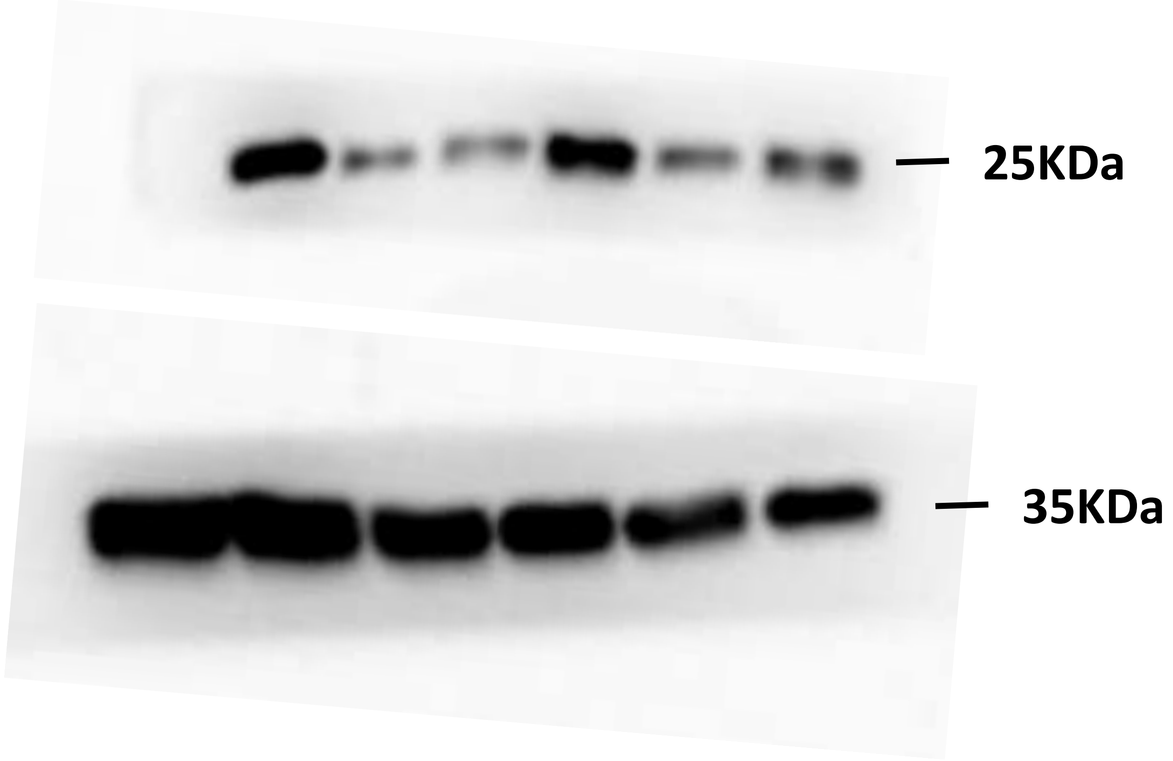

Fig. 5B-C

Raw data

B

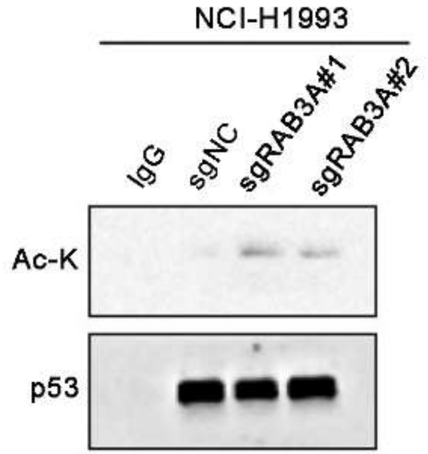

C

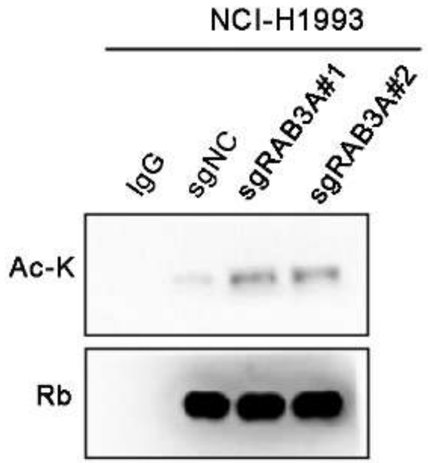

Fig. 5B

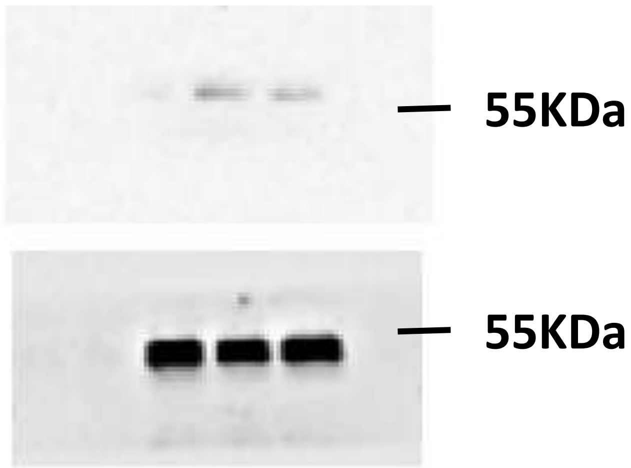

Fig. 5C

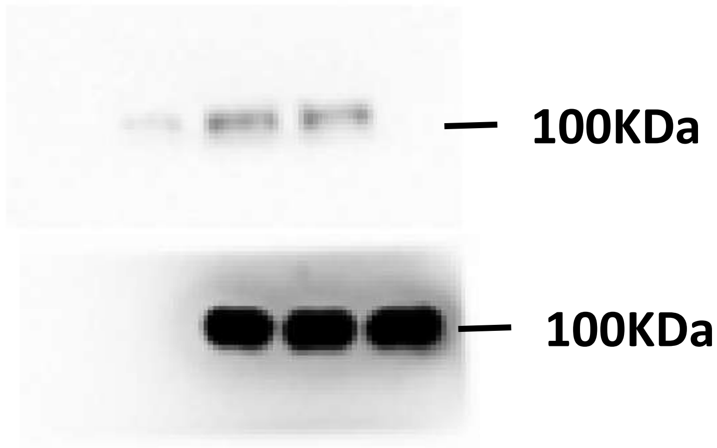

Fig. 5D

Raw data

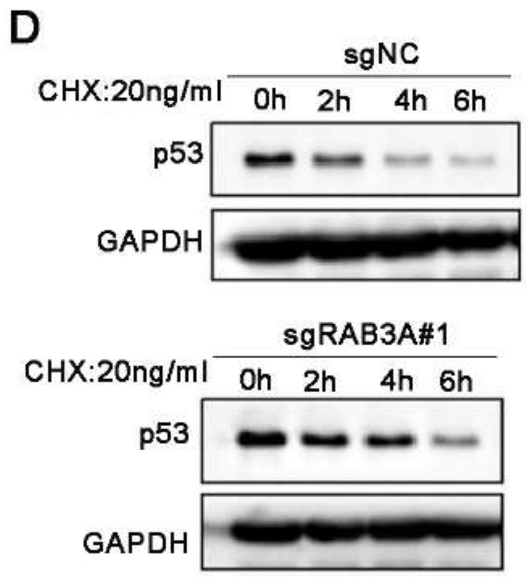

Fig. 5D

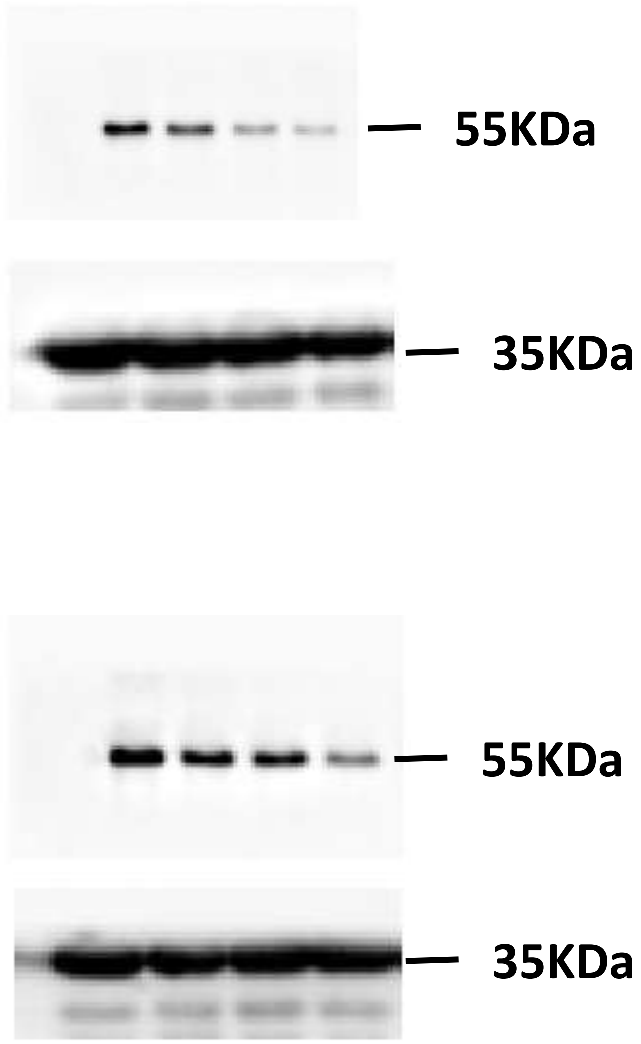

Fig. 5F

Raw data

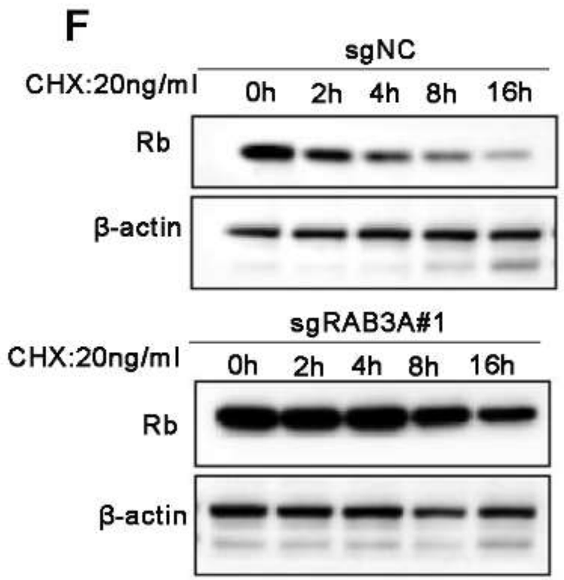

Fig. 5F

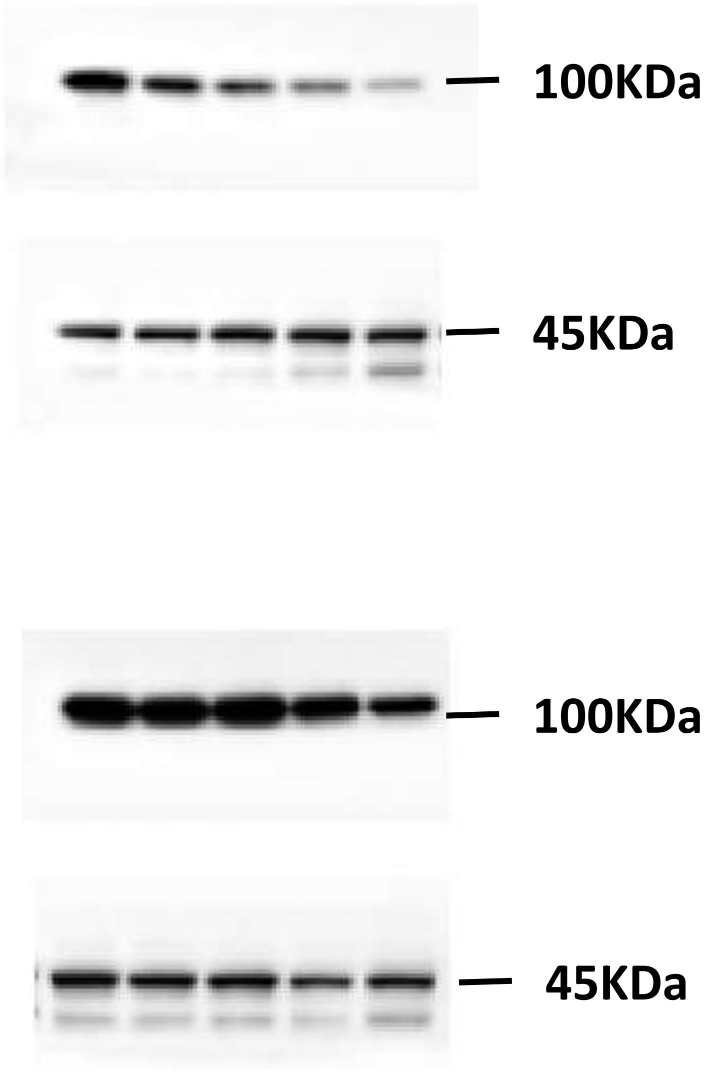

Fig. 5H-I

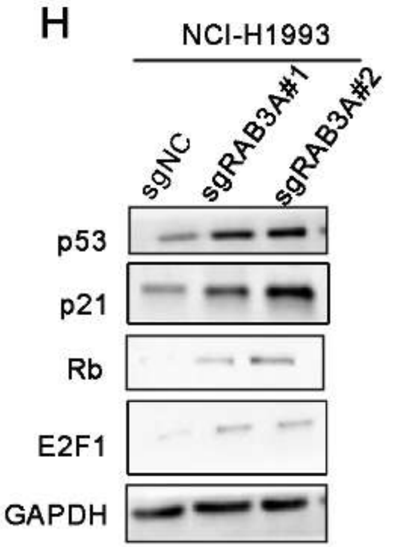

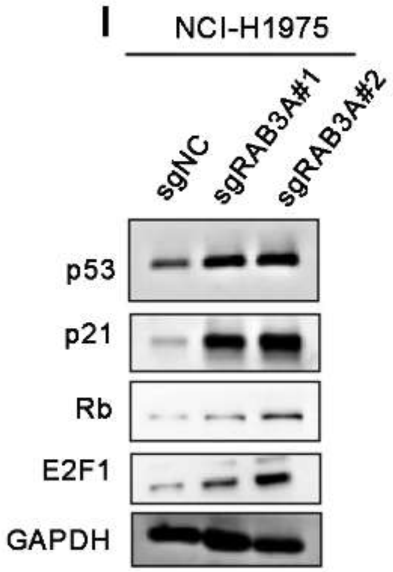

Raw data

Fig. 5H

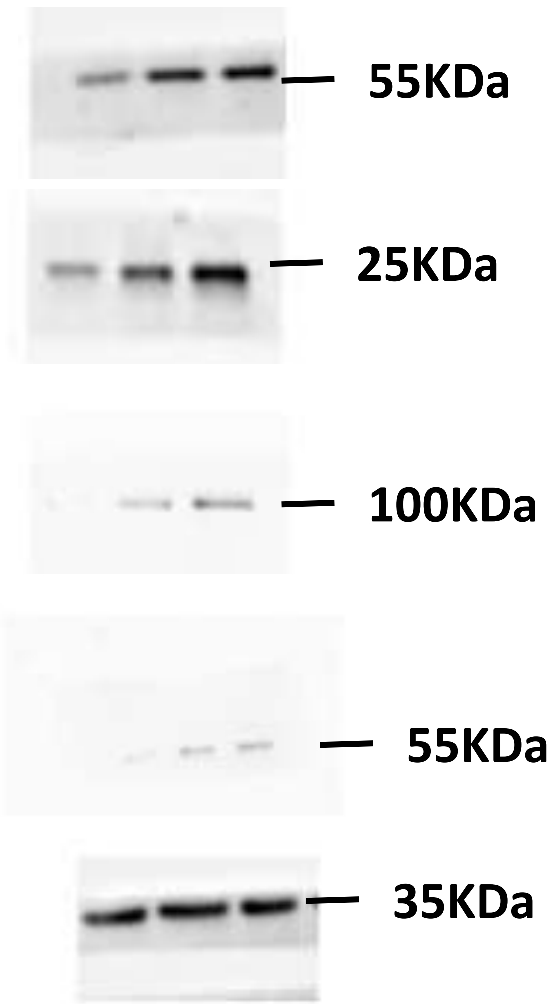

Fig. 5I

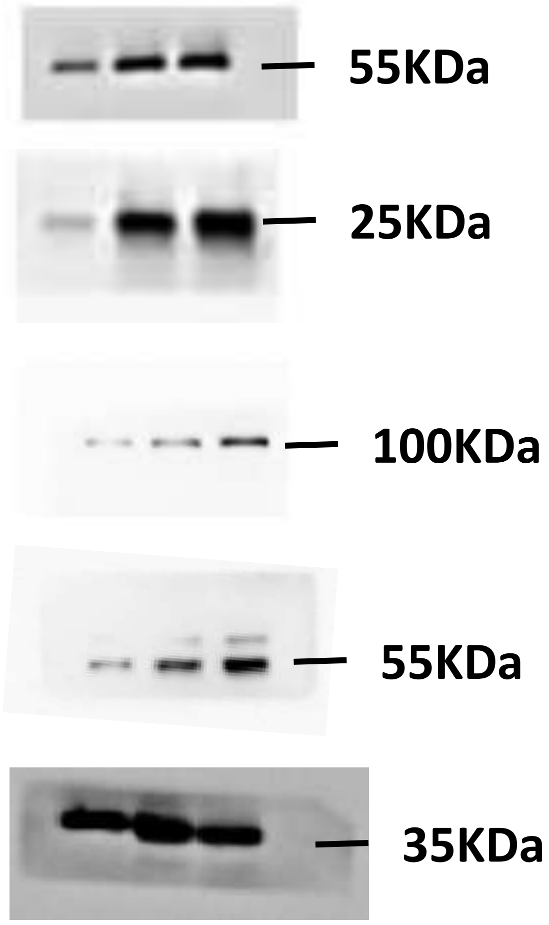

Fig 6.C-D

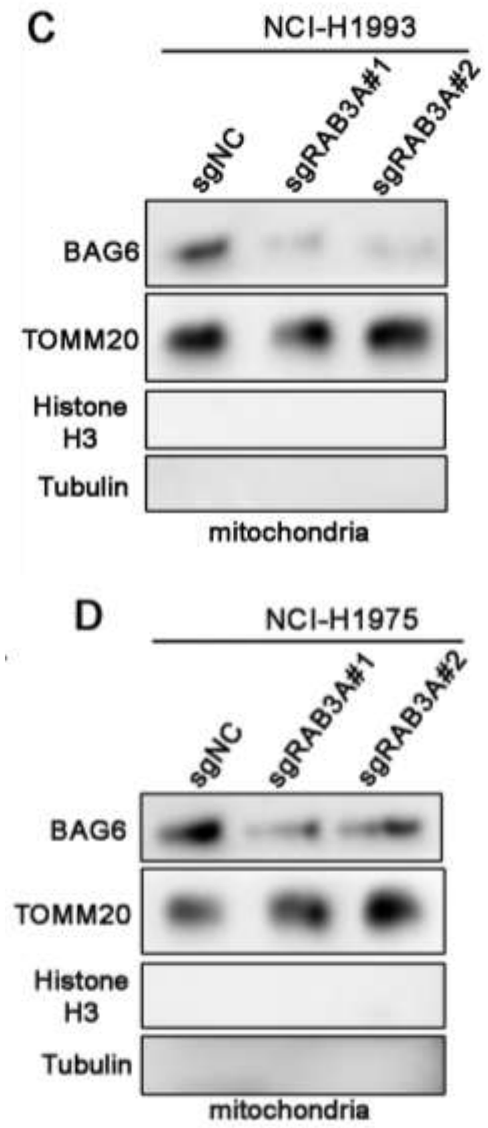

Raw data

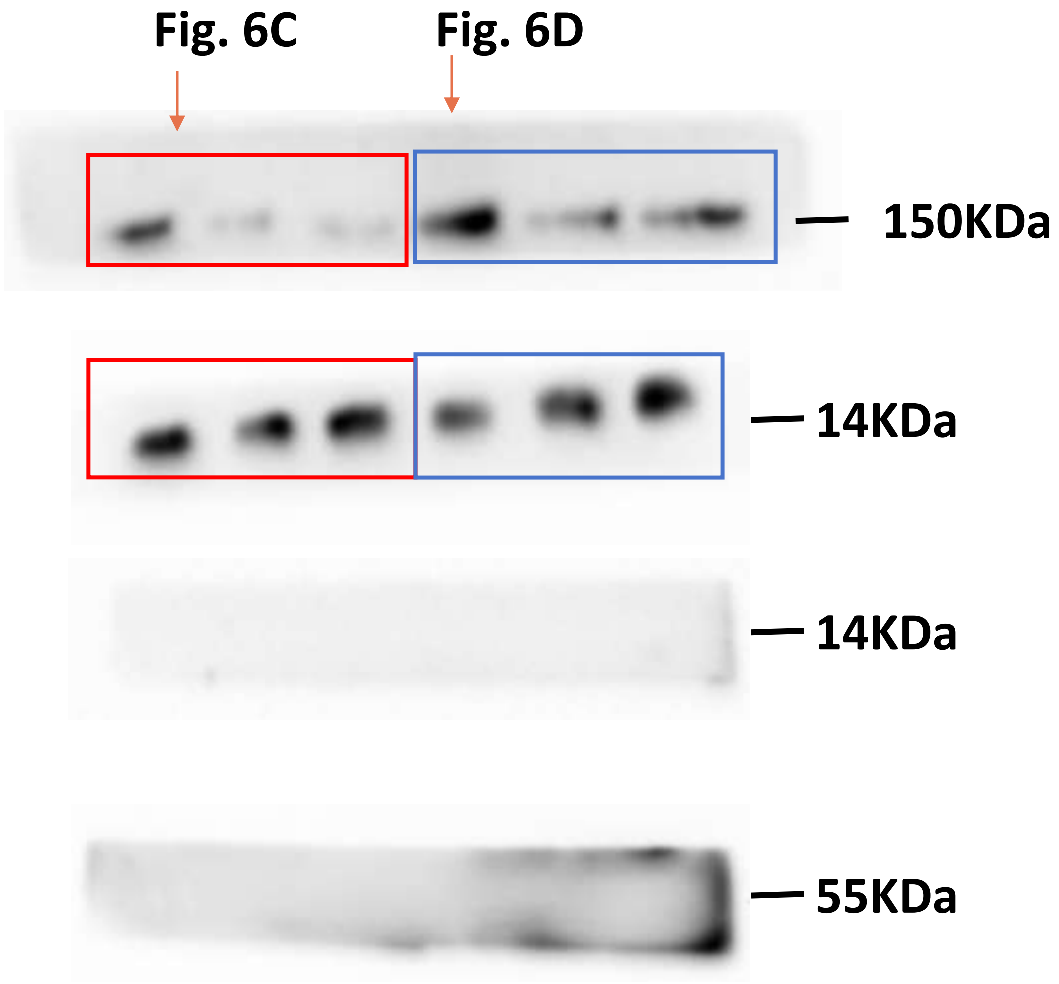

Fig. 6E

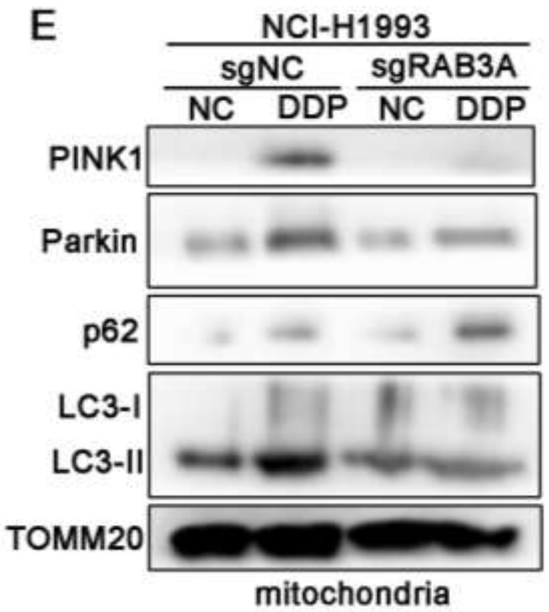

Raw data

Fig. 6E

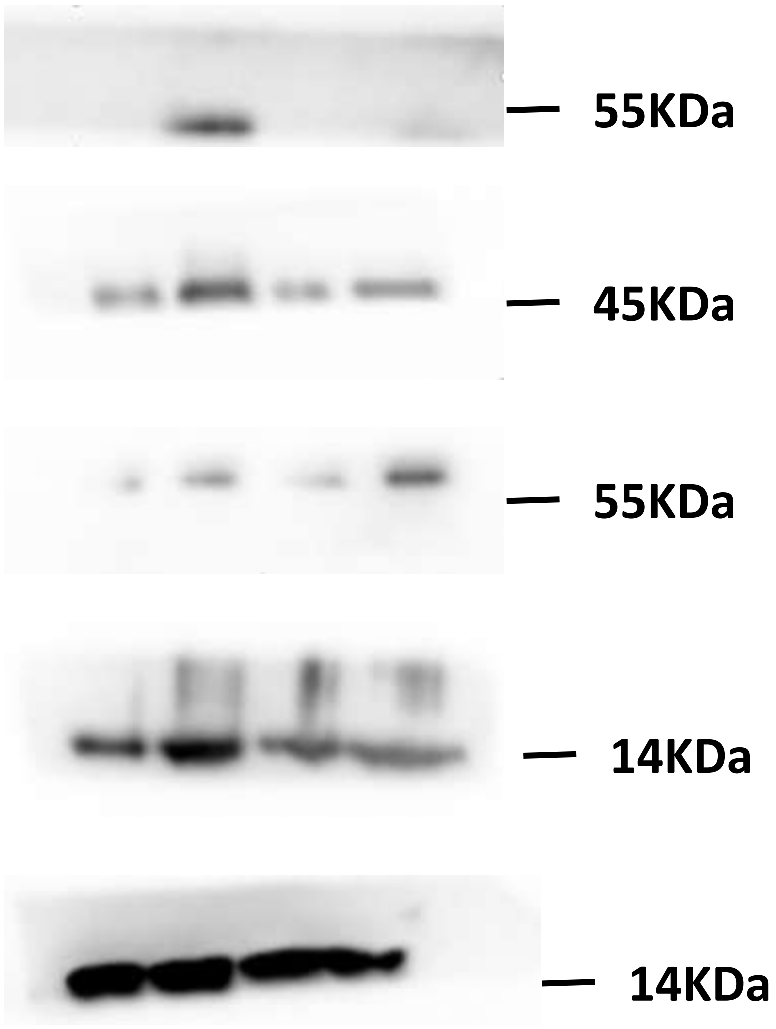

Fig. 6F

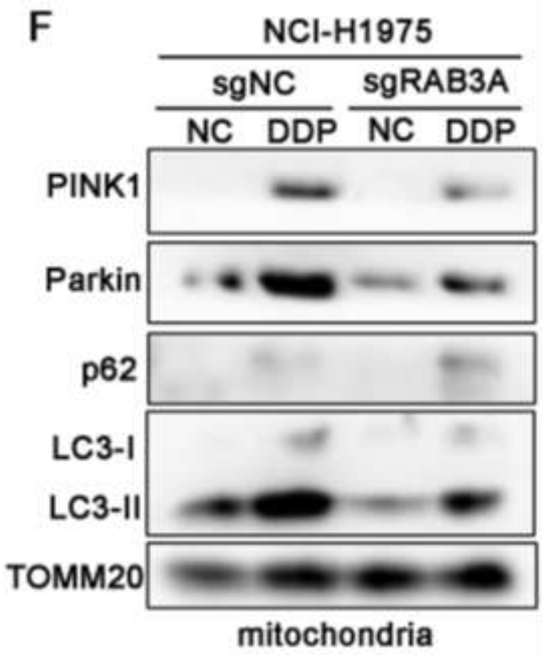

Raw data

Fig. 6F

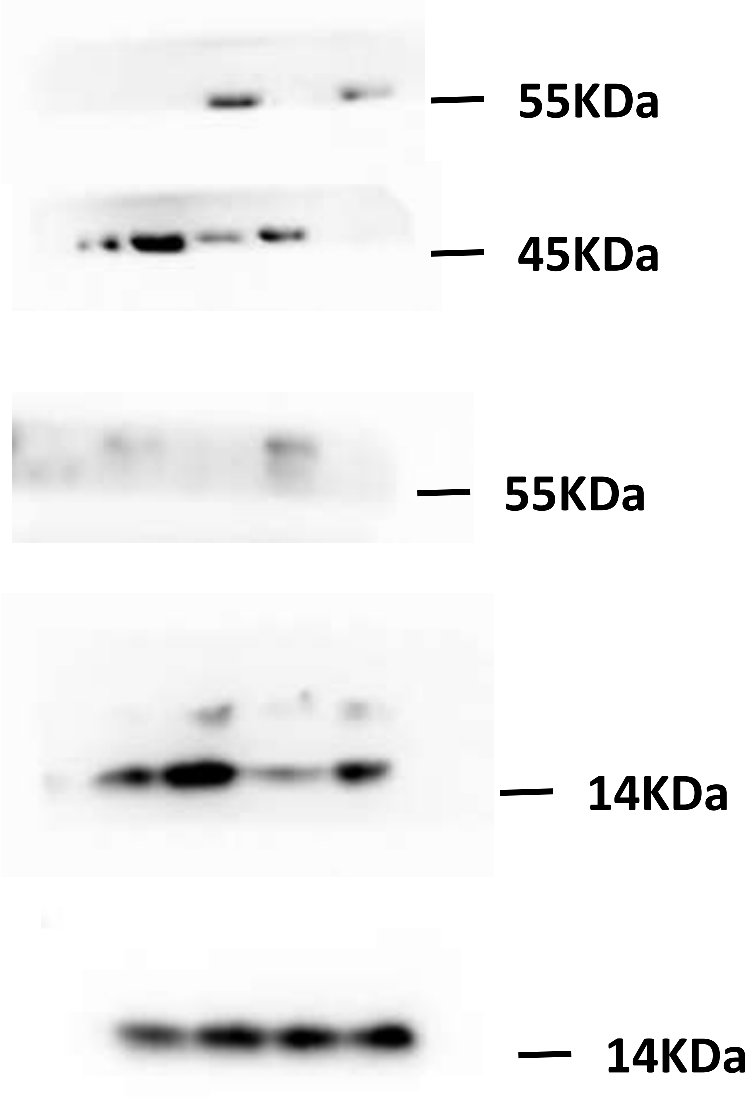

Fig. S1A

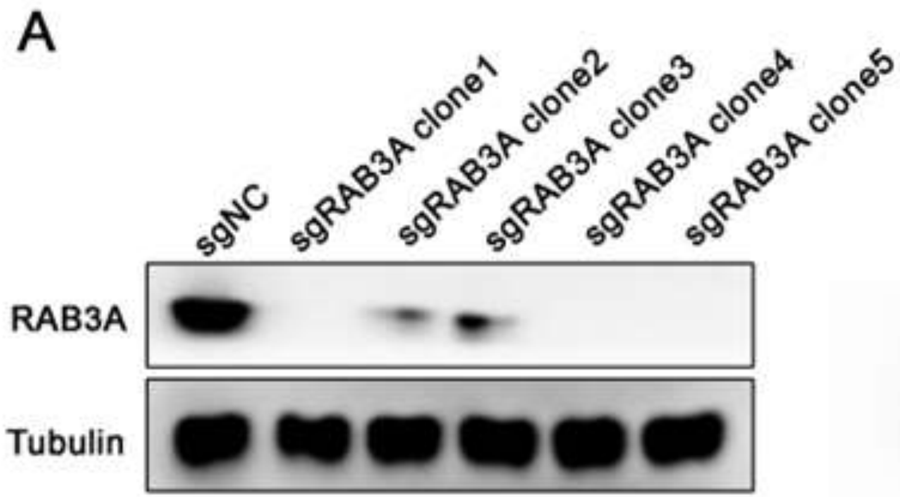

Raw data

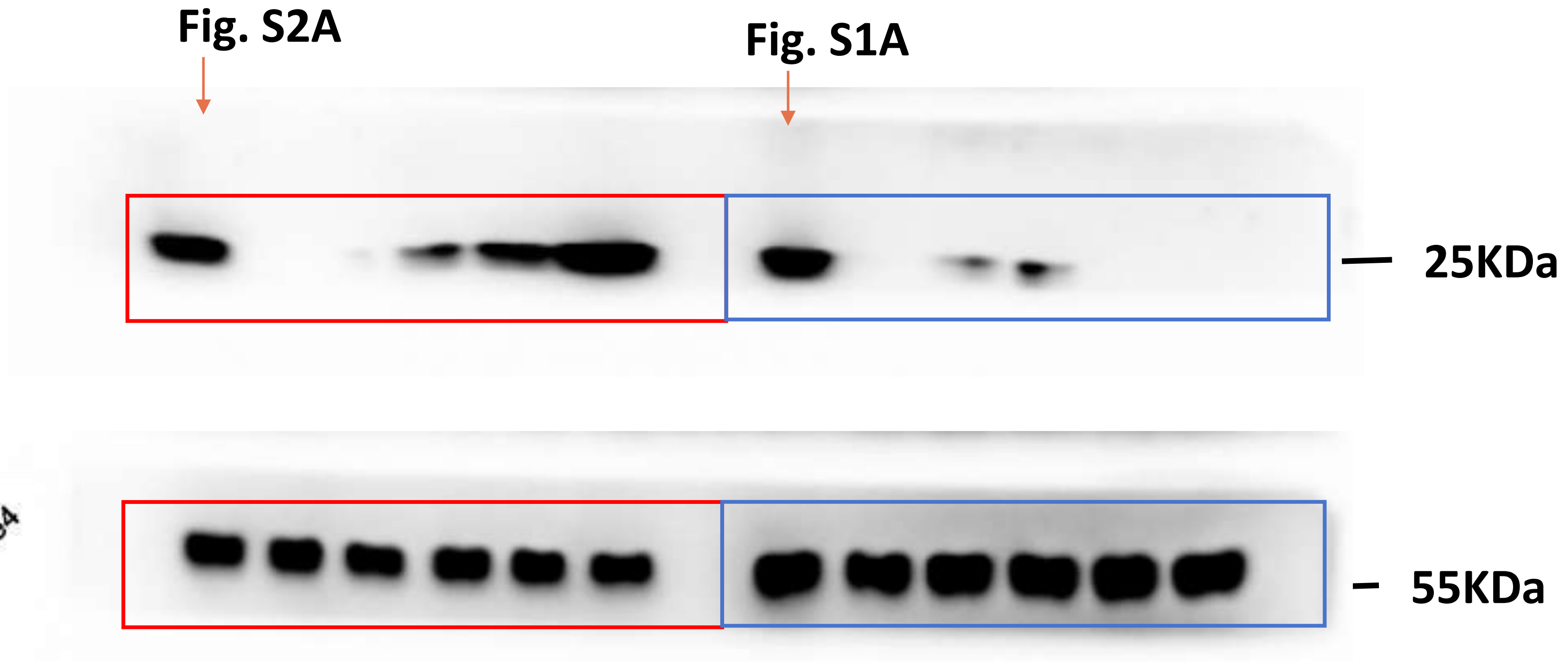

Fig. S2A

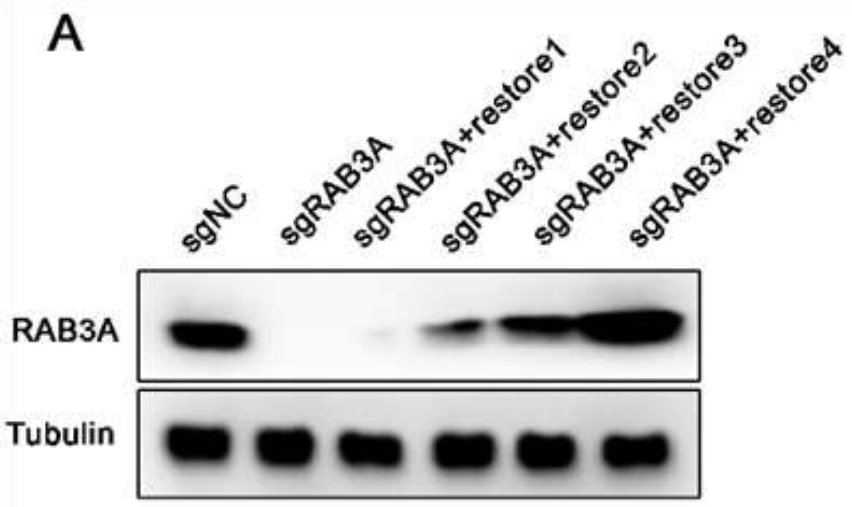

Fig. S5A

Raw data

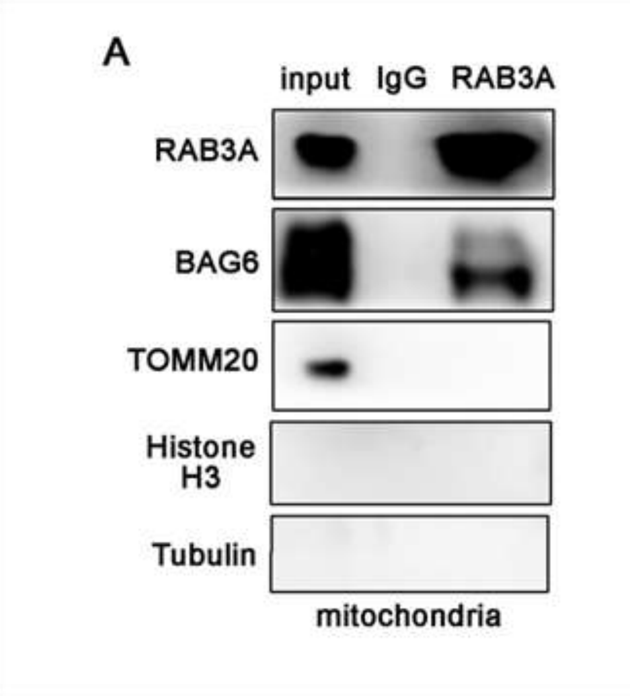

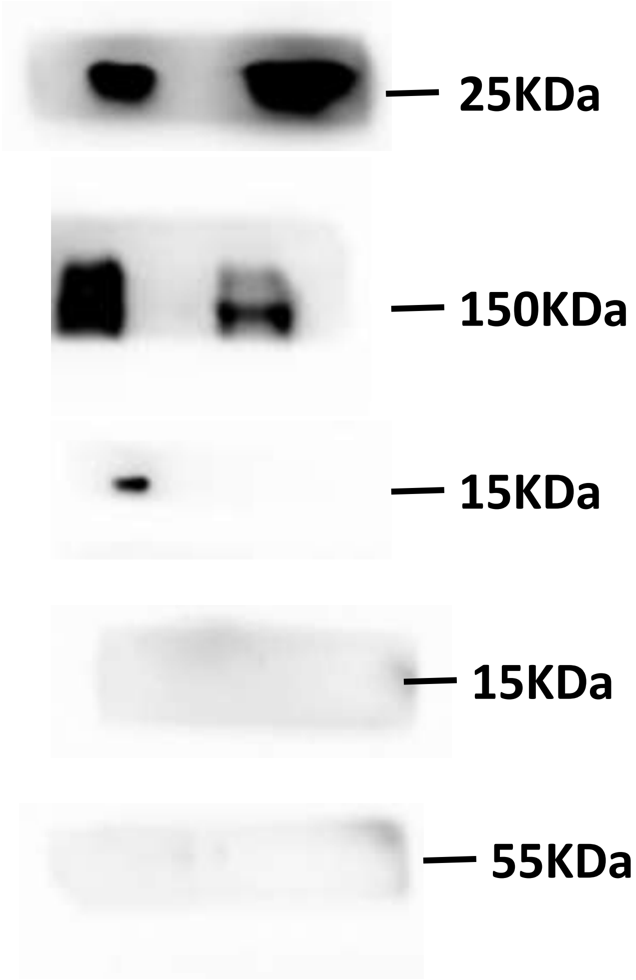

Fig. S5B

Raw data

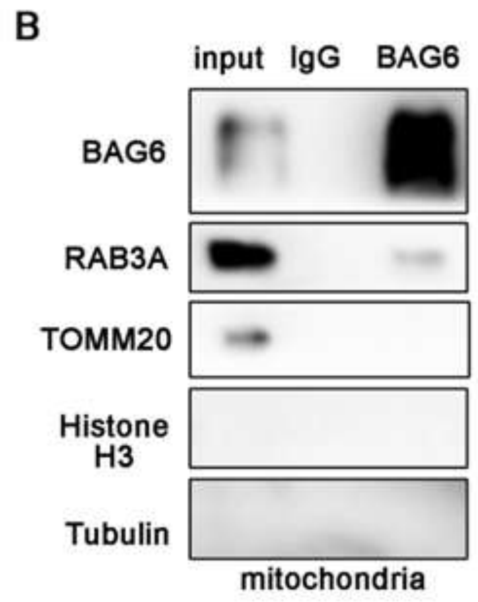

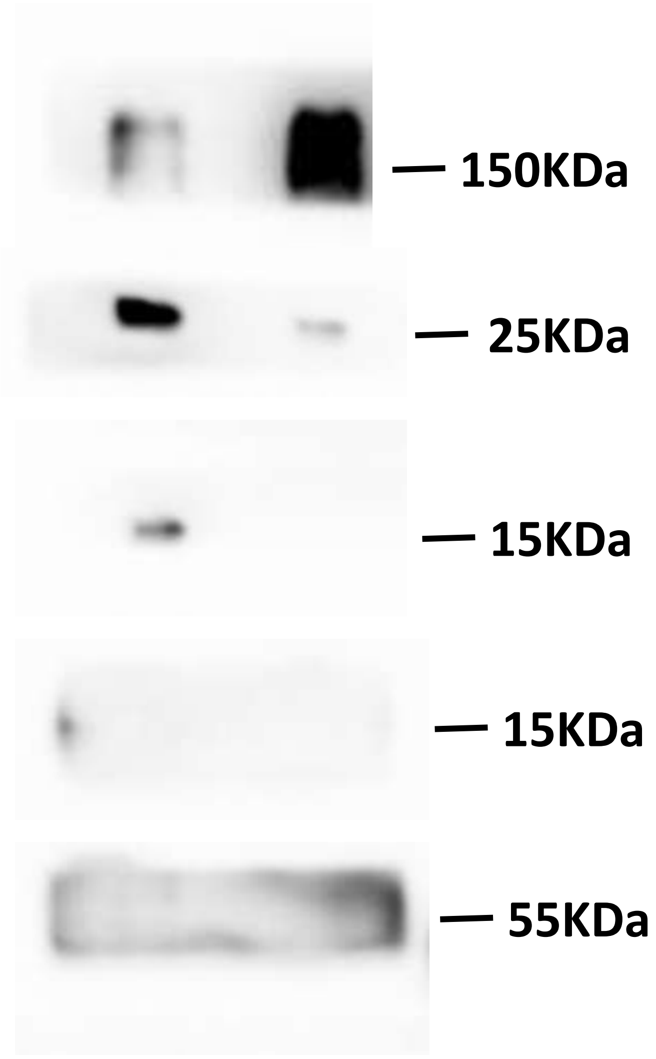

Fig. S6A

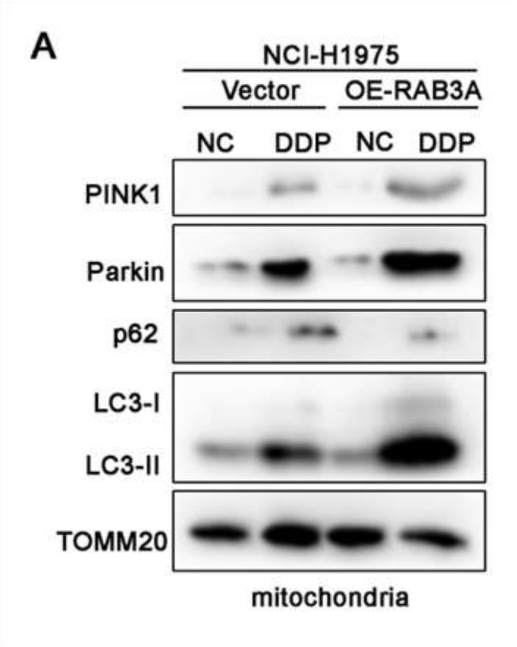

Raw data

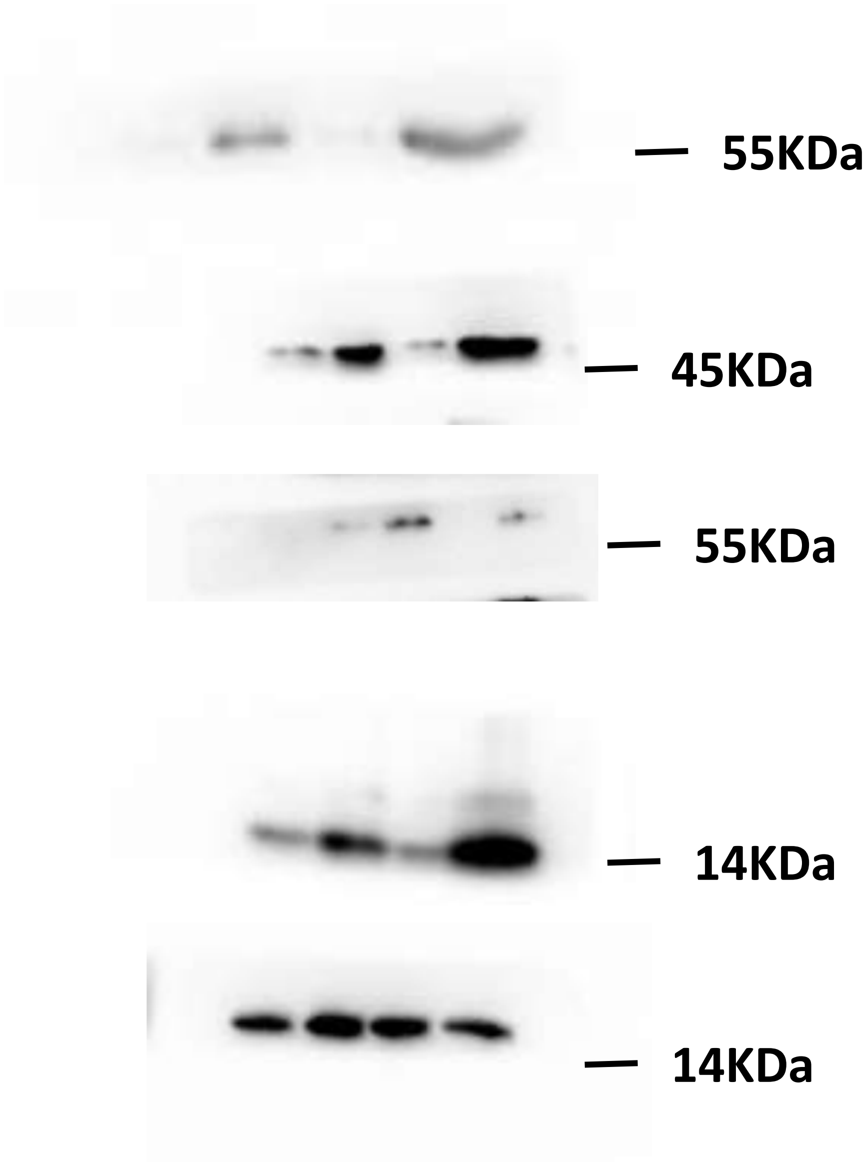

Fig. S7

Raw data

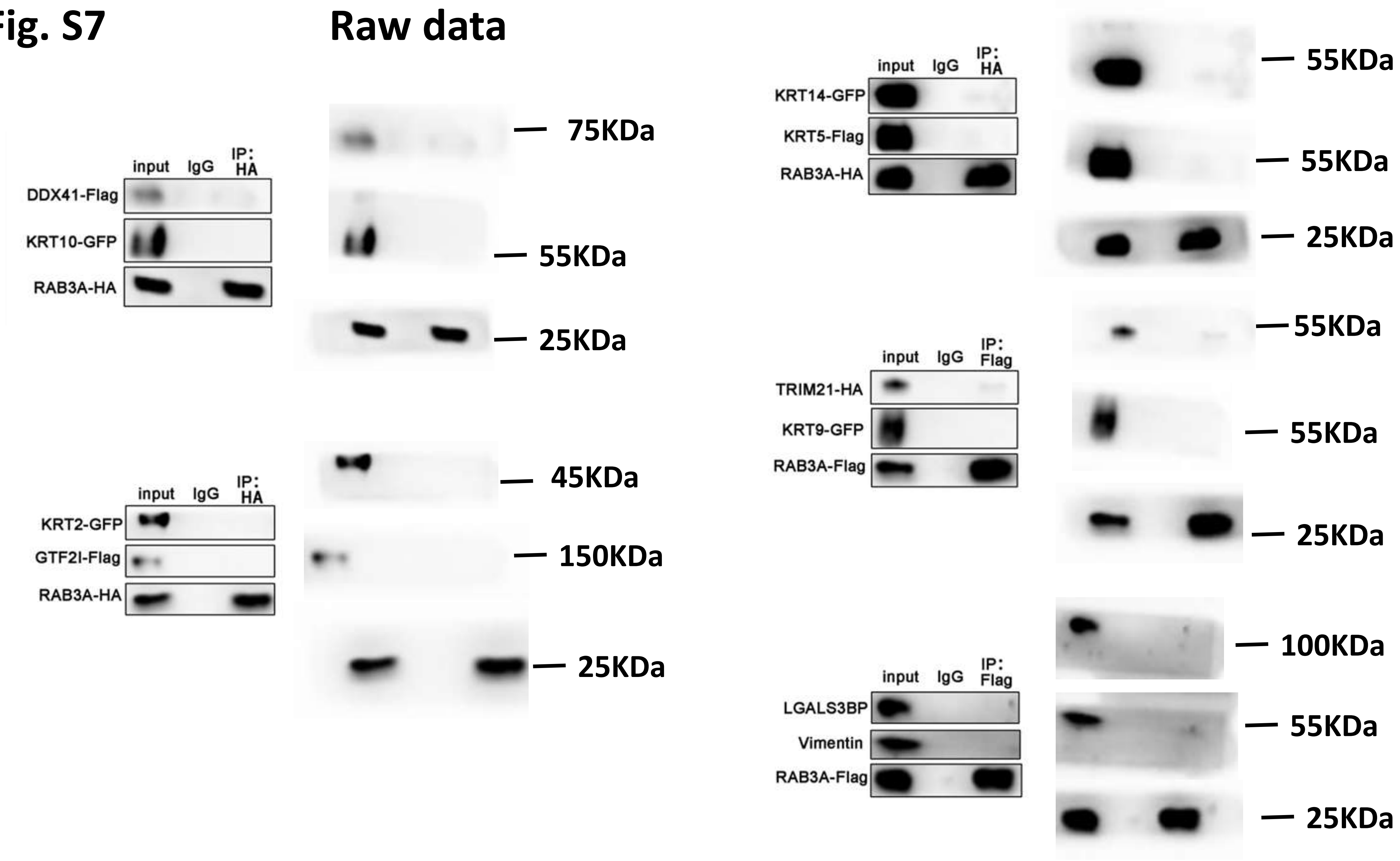

Fig. S8

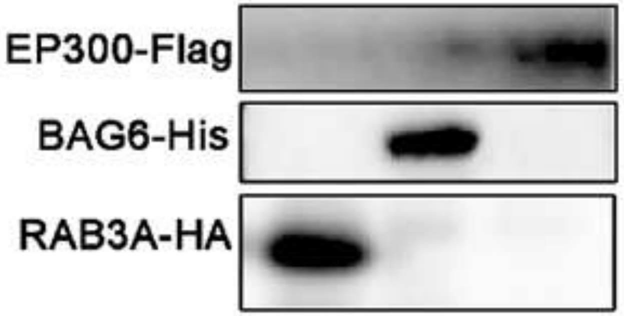

Raw data

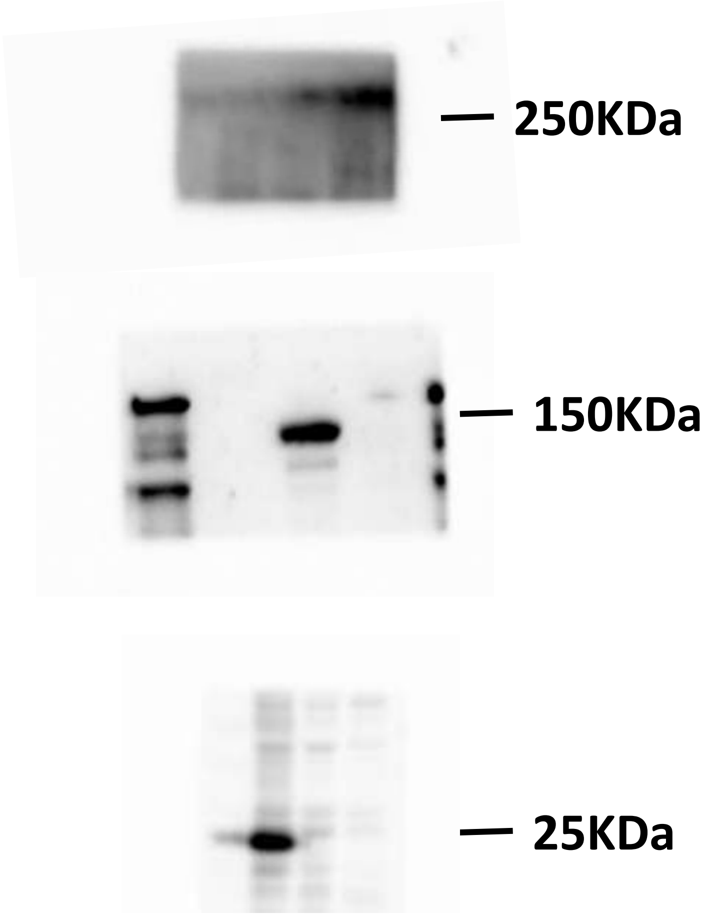

Supplement: Supplementary file 5 — Supplementary Material 5 [file 13402_2025_1123_MOESM5_ESM.pdf]
